# Human axillary lymph node T follicular helper (Tfh) and Precursor‐Tfh cells exhibit functional flexibility following seasonal influenza vaccination

**DOI:** 10.1002/cti2.70056

**Published:** 2025-10-23

**Authors:** Hannah Law, Raymond H. Y. Louie, Omid R. Faridani, Alexandra Carey Hoppé, Mollie Boyd, Mengfei Chen, Jerome Samir, Solange Obeid, Brad Milner, Fabio Luciani, Anthony D. Kelleher, Vanessa Venturi, C. Mee Ling Munier

**Affiliations:** ^1^ Immunovirology and Pathogenesis Program, Kirby Institute UNSW Sydney Sydney NSW Australia; ^2^ School of Computer Science and Engineering UNSW Sydney Sydney NSW Australia; ^3^ Garvan Institute of Medical Research Darlinghurst NSW Australia; ^4^ School of Biomedical Sciences UNSW Sydney Sydney NSW Australia; ^5^ St Vincent's Hospital Sydney Darlinghurst NSW Australia

**Keywords:** CD4+ T cells, fine needle aspiration, fine needle biopsy, human lymph nodes, influenza vaccination, T follicular helper cells

## Abstract

**Objectives:**

CD4^+^ T cells partitioned into different cell lineages based on transcription factor, cytokine and chemokine expression have defined functions that work cooperatively to ensure optimum operation of the immune system. This study investigated the lineages and interactions of key CD4^+^ T‐cell subsets within human lymph nodes (LNs) post‐vaccination to assess the potential for adaptability and flexibility in an immune response.

**Methods:**

Ultrasound‐guided fine needle biopsies/aspirates were used to isolate T follicular helper (Tfh) and Precursor (Pre)‐Tfh cells from the vaccine‐draining and contralateral axillary LNs of five individuals at 5 days after influenza vaccination, followed by single‐cell RNA sequencing, gene expression and T‐cell receptor analysis.

**Results:**

Tfh and Pre‐Tfh cells within five clusters with distinct gene expression profiles, designated: Resting, Activated migrating, B‐cell‐interacting Tfh, Proliferating and Cytotoxic, exhibited attributes of more than one T‐cell lineage and expanded T‐cell clones were present in more than one cluster, suggesting divergent differentiation into different fate lineages from a common precursor. Inferred pseudotime suggested a bifurcating trajectory rooted in the resting cluster and terminating at either the Proliferating or Cytotoxic clusters. This analysis predicted the transition of cells through activation states and the gain and loss of lineage attributes and effector functions. Enriched gene pathways along the pseudotime trajectories were consistent with these functional transitions and involvement in the immune response to vaccination.

**Conclusion:**

These results reveal the flexible potential within the Tfh lineage that could be leveraged to drive more efficient vaccine responses and inform rational vaccine design.

## Graphical abstract

Traditionally, the CD4^+^ T‐cell compartment has been partitioned into lineages based on transcription factor, cytokine and chemokine expression, with each distinct lineage having a critical and defined function. This study utilised fine needle biopsy to isolate key CD4^+^ T‐cell subsets from human lymph nodes following seasonal influenza vaccination to assess the potential for adaptability and flexibility in these lymph node cell populations. Analysis of single‐cell transcriptomic data revealed five functionally distinct clusters and inferred pseudotime analysis was used to construct a differentiation trajectory revealing the transition of cells through activation states and gain and loss of different lineage attributes and effector functions.

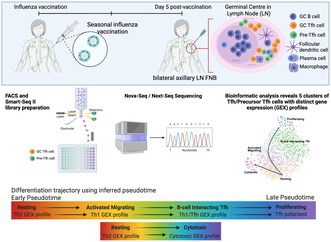

## Introduction

Traditionally, the CD4^+^ Tcell compartment is partitioned into lineages according to a cell's expression of transcription factors, cytokines and chemokines. Much like cogs in a machine, each distinct lineage has a critical and defined function that must work collaboratively to underpin the immune system's ability to control and eliminate the vast array of pathogens encountered by a host.^1^ An increasing number of studies have demonstrated the adaptability of CD4^+^ T cells, where changes in inflammatory milieu or pathogenic stimuli result in varying degrees of phenotypic plasticity or repolarisation towards a mixed phenotype.^2^, ^3^, ^4^, ^5^, ^6^, ^7^, ^8^, ^9^, ^10^ The rise in popularity and accessibility of single‐cell transcriptomics has further revealed an impressive capacity for heterogeneity within the CD4^+^ Tcell compartment.^11^, ^12^, ^13^, ^14^, ^15^ Most studies have utilised animal models or human peripheral blood. However, more recently, studies have begun to focus on the human lymph nodes (LNs),^14^, ^15^, ^16^ because of the unique LN microenvironment where cellular phenotype and dynamics are heavily influenced by stimuli external to the cell.

Within the LN, T follicular helper (Tfh) cells have been established as key players in effective immune responses because of their critical role in the establishment and maintenance of the germinal centre (GC) reaction via delivery of specialised help to B cells. Following migration of naïve CD4^+^ T cells into the LNs and priming with antigen in the T‐cell zone, downregulation of CCR7 is complemented by upregulation of CXCR5, promoting homing towards the B‐cell follicle.^17^, ^18^ It is here, on the periphery of the B‐cell follicle, that cognate interactions between Precursor Tfh (Pre‐Tfh) and B cells occur, promoting further differentiation and sealing the Tfh cell fate.^19^ A subpopulation of Tfh cells participates in the formation of the GC and fulfil their role as essential B‐cell helpers, influencing the degree of somatic hypermutation and affinity maturation of GC B cells, ultimately shaping the quality of the humoral response.^20^, ^21^

In mouse models, Pre‐Tfh cells that remain in the follicular mantle of the LN and do not form part of the GC reaction can be rapidly reactivated upon antigen encounter, contributing to seeding the memory pool with antigen‐responsive Tfh cells that perform effector functions outside of the GC^22^ and potentially establishing a circulating‐Tfh (c‐Tfh) cell population in the periphery.^23^ Another recent mouse model described the differentiation of Pre‐Tfh cells with two potential fates: continuation along the Tfh differentiation pathway to form the mature GC Tfh population and seeding a PD‐1^low^ memory precursor phenotype population.^24^ These results were further supported by Feng *et al*.,^25^ who demonstrated a subpopulation of PD‐1^low^ expressing Tfh cells, consistent with the phenotypic definition of Pre‐Tfh cells presented here, that exhibited increased survival and recall response capacity. Despite the growing body of evidence that Pre‐Tfh cells residing outside of the GC contribute to a memory population with the capacity for rapid antigen responses, further investigation is required to elucidate the mechanisms by which this occurs. Although GC Tfh cells have a defined differentiation pathway, the Th1 and Th2 gene programmes have been shown to be imprinted on a proportion of these cells in animal models.^26^, ^27^ Considerable research on the sub‐lineages of c‐Tfh cells has contributed to our understanding of the functional diversity of the Tfh population in peripheral blood. Th1‐like, Th2‐like and Th17‐like c‐Tfh cells have been identified, each with varying abilities to provide help to B cells.^28^, ^29^

Recent studies using single‐cell RNA sequencing (scRNA‐seq) have demonstrated that vaccination can induce dynamic and transcriptionally diverse Tfh cell populations in the draining LN.^14^, ^15^ However, there is a dearth of knowledge about the functional flexibility of the Tfh cell population within the human LN. Three potential models of Tfh cell plasticity have been discussed in the literature^30^: (1) a partial alternate Th programme is imprinted onto Tfh cells during initial direct Th‐specific antigenic cell priming of T cells; (2) a Th1, Th2 or Th17 inductive environment generated during initial direct T‐cell priming may activate partial gene expression programmes in bystander Tfh cells; and (3) the Pre‐Tfh population represents a less‐polarised or fixed‐fate population that is more likely to express attributes of more than one lineage. Conventionally, cell types are subset based on cytokine production; however, Tfh cell production of cytokines is limited,^31^, ^32^ potentially to avoid non‐specific activation of bystander cells within the LN because of the GC reaction.^33^ Therefore, the transcriptome represents a powerful tool in delineating the cell subsets and their potential for functional flexibility within the Pre‐Tfh and GC Tfh differentiation pathway. Additionally, the combination of gene expression and T‐cell receptor (TCR) data yielded from scRNA‐seq allows identification of clonally related cells with different gene expression profiles. Understanding the potential for the functional flexibility of Pre‐Tfh and GC Tfh cell populations would greatly inform rational vaccine design, representing a possible target to guide the immune response towards a desired lineage or effector function.

In our previous study^34^ of the early secondary immune response dynamics of the GC Tfh and Pre‐Tfh cell populations, we examined the immunophenotype of these cell populations, obtained by ultrasound‐guided fine needle biopsy (FNB) of the axillary LNs pre‐vaccination (baseline) and at Day 5 following seasonal influenza vaccination in healthy individuals with previous exposure to influenza infection or vaccination. We reported significant increases in the numbers of Tfh, but not Pre‐Tfh, cells in the draining LNs between the baseline and Day 5 post‐vaccination timepoints. However, between these timepoints we observed significant increases in the frequency of both activated (CD38^+^ICOS^+^) and activated and proliferating (CD38^+^Ki67^+^) GC Tfh and Pre‐Tfh cells. In this study, we elucidate the functional divisions within the Tfh cell population from human axillary LNs during the early phase of the secondary immune responses to seasonal influenza vaccination. For five of the participants from our previous study, we used cells from the Day 5 post‐vaccination FNB samples that were index sorted and prepared for scRNA‐seq to investigate the heterogeneity and differentiation pathways post‐vaccination, with the primary aim of interrogating the subtle ‘tug‐of‐war’ between CD4^+^ T‐cell lineage programmes during Tfh cell differentiation. Transitions in activation state and effector functions throughout the Tfh differentiation programme in response to vaccination were observed using pseudotime trajectory analysis.

## Results

### Clusters with distinct gene expression profiles and attributes of multiple CD4 ^+^ T‐cell lineages are composed of both Tfh and Pre‐Tfh cells

The existence of Tfh cells with multiple partial CD4^+^ T‐cell lineage phenotypes within the LN is an exciting hypothesis as it would form the foundation of functional distinctions that could be leveraged to bias the immune response to vaccination towards a desired phenotype. To characterise the functional subsets that exist within human LN derived Pre‐Tfh and GC Tfh cell populations, we used the FNB technique to isolate these populations of interest from draining and contralateral axillary LNs of five vaccinated healthy volunteers during a secondary immune response, at 5 days following seasonal influenza vaccination. Index sorted GC Tfh cells were defined as CD3^+^CD4^+^CD45RA^−^PD‐1^high^CXCR5^high^ and Pre‐Tfh cells were defined as CD3^+^CD4^+^CD45RA^−^PD‐1^+^CXCR5^+^ (Supplementary figure 1). scRNA‐seq was performed on four cell populations (draining/contralateral LN and Tfh/Pre‐Tfh) per participant. The details of the study cohort and vaccination, as well as the numbers of cells sequenced per participant, are summarised in Supplementary table 1.

**Figure 1:**
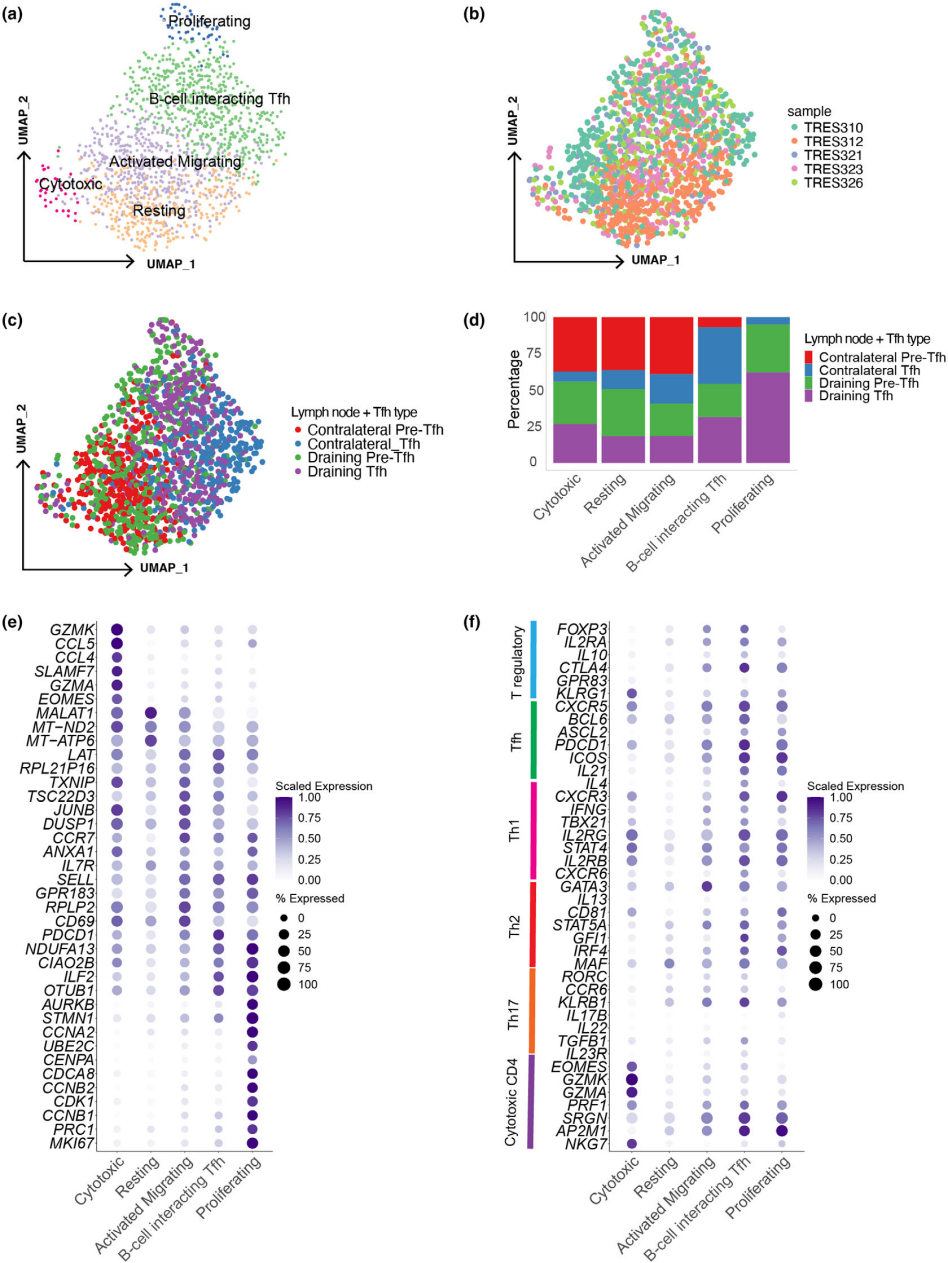
Phenotypically distinct clusters exhibit attributes of multiple CD4^+^ T‐cell lineages. **(a)** Uniform Manifold Approximation and Projection (UMAP) dimensionality reduction and clustering reveals five distinct clusters that were subsequently annotated as: Resting (311 cells), Activated Migrating (524 cells), B‐cell‐interacting T follicular helper (Tfh) (579 cells), Proliferating (51 cells) and Cytotoxic (34 cells). UMAP with cells coloured by **(b)** participant and **(c)** the sorted cell population [i.e. contralateral or draining lymph nodes (LN) and Tfh or Precursor Tfh (Pre‐Tfh)]. **(d)** Bar plot of the sorted cell population contribution to each of the five clusters. **(e)** Dot plot of cluster‐defining genes. Size of dots represents percentage of cells within a cluster expressing a given gene and colour represents scaled expression values. **(f)** Dot plot of an *a priori* gene list for the major CD4^+^ T‐cell lineages.

Dimensionality reduction, using Uniform Manifold Approximation and Projection (UMAP), and cluster analysis, based on differential gene expression (DGE), of the 1499 cells in the post‐quality control dataset identified five clusters with distinct gene expression profiles (Figure 1a). The numbers of cells with gene expression data per sample and per cluster are summarised in Supplementary table 3. Cells from the various participants and sorted cell populations were distributed across the UMAP (Figure 1b and c). However, sorted Tfh cells tended to dominate the right‐hand side of the UMAP, while Pre‐Tfh cells tended to dominate the left‐hand side of the UMAP (Figure 1c), which was compatible with the relative expression of canonical gene markers associated with Tfh and Pre‐Tfh cells, such as *BCL6*, *PDCD1* (PD‐1) and *CXCR5* (Supplementary figure 2a).

The five clusters of Tfh and Pre‐Tfh cells were annotated, using the differentially expressed gene profiles, as: Resting (homeostasis and cell cycle regulation genes; *MALAT1*, *MT‐ND2* and *MT‐ATP6*), Activated Migrating (genes required for migration within the LN; *SELL*, *CCR7* and *GPR183* and activation genes; *LAT*, *ANXA1* and *CD69*), B‐cell‐interacting Tfh (LN homing genes; *SELL* and *GPR183* and T‐cell anergy marker *OTUB1*), Proliferating (proliferation and cell cycle genes; *MKI67* (Ki67), *UBE2C*, *CENPA*, *CDCA8*, *CCNA2*, *CCNB1*, *CCNB2* and *PRC1*) and Cytotoxic (cytolytic genes; *GZMK*, *CCL5*, *CCL4*, *SLAMF7*, *GZMA* and *EOMES*) clusters (Figure 1e). The expression of the differentially expressed genes per cluster for the index sorted Pre‐Tfh and Tfh cell populations is shown in Supplementary figure 2b and details of each gene function can be found in Supplementary table 4. All clusters, excluding the Proliferating cluster, were formed by cells from both draining and contralateral LNs and Pre‐Tfh and Tfh populations (Figure 1d). Notably, the Proliferating cluster was formed predominantly by draining LN cells.

To determine whether the identified five clusters of Tfh and Pre‐Tfh cells corresponded to canonical CD4^+^ T‐cell lineages, we investigated the expression of an *a priori* gene list representing the different lineages for all cells (Figure 1f) and for the sorted Pre‐Tfh and Tfh cells (Supplementary figure 2c) per cluster. The Cytotoxic and Resting clusters expressed no or very low levels of regulatory T‐cell (Treg) associated genes. The remaining clusters expressed moderate to high levels of Treg genes (excluding *GPR83*), therefore exhibiting a partial Treg gene profile (Figure 1f, *FOXP3* in Supplementary figure 2a). Three clusters, Activated Migrating, B‐cell‐interacting Tfh and Proliferating, displayed key characteristics of the Tfh cell lineage, expressing moderate to high levels of canonical markers *CXCR5*, *PDCD1* (PD‐1), *IL21* and *ICOS*, and the major transcription factor *BCL6*. Whereas the Cytotoxic and Resting clusters expressed low levels of Tfh associated genes. Most widely expressed was the Th1 lineage gene expression profile, with all clusters, excluding the Resting cluster, expressing moderate levels of most Th1 associated genes in a high proportion of cells. All clusters had moderate expression of key Th2 genes *STAT5A* and *MAF*, the major transcription factor *GATA3*, low levels of expression of *RORC*, *CCR6, TGFB1* and a range of moderate to high expression of *KLRB1*, suggesting Th17 is not an established functional subset within these LN cells. The Cytotoxic cluster exhibited high scaled expression of key cytotoxic genes, whereas the remaining clusters had low expression of *EOMES*, *GZMK*, *GZMA* and *NKG7*. Interestingly, these expression profiles were reversed for *SRGN* and *AP2M1*, suggesting these genes could have other functional roles. Taken together, these results demonstrate that Tfh and Pre‐Tfh cells have multiple CD4^+^ T‐cell lineage gene expression profiles contributing to the composition of each cluster, suggesting these cell populations may be highly functionally diverse.

### T‐cell clonality suggests functional flexibility of Pre‐Tfh and Tfh cells

**Figure 2:**
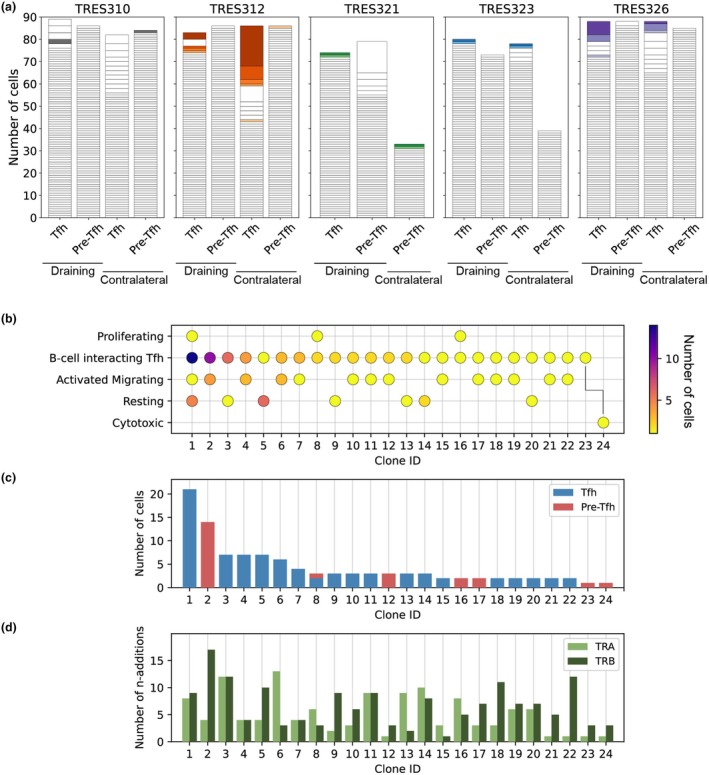
T‐cell receptor(TCR) clones present in multiple clusters suggest T‐cell flexibility. **(a)** The clonal structure of the TCRɑβ repertoires for each sample. Each segment of the bar plots represents a TCRɑβ and the size of the segment indicates the number of cells with that TCRɑβ. Colour segments indicate TCRɑβ, identical at the level of the nucleotide sequence, that were common across samples for each participant. There were no shared TCRɑβ observed between participants. **(b–d)** TCRɑβ clones were observed in multiple clusters with different effector phenotypes. **(b)** TCRɑβ clones, with identical V and J gene usage and CDR3 nucleotide sequence, that were observed across more than one cluster. The dots indicate the clusters in which each TCRɑβ clone was observed, with the number of cells per cluster indicated by the colour map. Clones 23 and 24 had TCRɑ that were identical at the level of the nucleotide sequence and TCRβ that were identical at the level of the amino acid sequence, with the CDR3β sequences differing by a single nucleotide, which could potentially be a sequencing error or an example of convergent recombination. **(c)** The contribution of cells from the sorted T follicular helper (Tfh) and Precursor Tfh (Pre‐Tfh) cell populations to each of the TCR clones. **(d)** The estimated minimal number of nucleotide additions (*n*‐additions) required for the generation of the TCRɑ and TCRβ for each TCR clone. TCR sequences with higher numbers of *n*‐additions tend to have relatively lower probabilities of generation by the V(D)J gene recombination process, suggesting that the cells comprising these T‐cell clones are more likely related by a common precursor than derived from multiple precursor cells.

To investigate the potential functional flexibility of the Pre‐Tfh and Tfh cells, as inferred by the transcriptomic gene expression profiles, we considered the distribution of clones across clusters using the TCR, which forms the cell's natural barcode. Here, we defined a T‐cell clone as the paired TCR α‐ and β‐chains, identified by their V and J gene usage, and CDR3 nucleotide sequence. The clonal composition of the TCRαβ repertoires for each LN sample per participant, as well as the TCRαβ clones common across samples per participant, are shown in Figure 2a. There were no TCRαβ clones shared across participants. When we examined the TCRαβ sequences of the cells contributing to each cluster, we identified 22 expanded T‐cell clones present in more than one cluster and that therefore exhibited attributes of more than one CD4^+^ T‐cell lineage (Figure 2b, Supplementary table 5). Two other clones, Clones 23 and 24, found within the B‐cell‐interacting Tfh and Cytotoxic clusters, respectively, had TCRɑ that were identical at the level of the nucleotide sequence and TCRβ that were identical at the level of the amino acid sequence. The CDR3β sequences of these two clones differed by a single nucleotide (Supplementary table 5), which could potentially be a sequencing error or an example of convergent recombination.^35^ With the exception of Clone 8, comprising cells from both the sorted Pre‐Tfh and Tfh populations, all copies of the T‐cell clones observed in more than one cluster originated from either the Pre‐Tfh or Tfh cell populations (Figure 2c). One of the largest clones identified in this dataset (Clone 1) spanned four clusters: the Resting, Activated Migrating, B‐cell‐interacting Tfh and Proliferating clusters, while the other clones were each observed in two clusters (Figure 2b). These cells, that have different gene expression profiles and share the same TCRαβ, may arise from a common precursor, whereby a cell with a particular TCRαβ proliferated and produced multiple cells with the same TCRαβ, which would demonstrate a divergent differentiation trajectory towards multiple cell lineages.^36^ Alternately, these cells could be derived from multiple precursor cells, each produced with an identical TCRαβ generated by an independent V(D)J gene recombination event. The generation of TCRαβ, identical at the level of the TCR nucleotide sequence, by multiple recombination events during the largely stochastic process of V(D)J recombination would require the TCRαβ to have a sufficiently high generation probability. There is an established association between the number of nucleotide additions required to produce a TCR by the gene recombination process and the probability of the TCR being generated, with TCR sequences that require many nucleotide additions tending to have a lower probability of being generated, and therefore less likely to be generated by multiple recombination events.^35^, ^37^, ^38^ Therefore, to investigate the possibility of the cells observed across multiple clusters having different precursors, we determined the minimum numbers of nucleotide additions required to generate the TCRα and TCRβ sequences of each clone. Most of the T‐cell clones observed in multiple clusters had TCRα and/or TCRβ sequences that required relatively high numbers of nucleotide additions (Figure 2d), suggesting lower generation probabilities, which supports the hypothesis that these cells do not have multiple precursors but rather shared a common precursor. Overall, these results suggest the potential functional flexibility, as inferred by the transcriptomic gene expression profiles, of Pre‐Tfh and Tfh cells post‐vaccination.

### TCRαβ that map to previously reported influenza‐specific TCR sequences were observed in all five clusters

**Figure 3:**
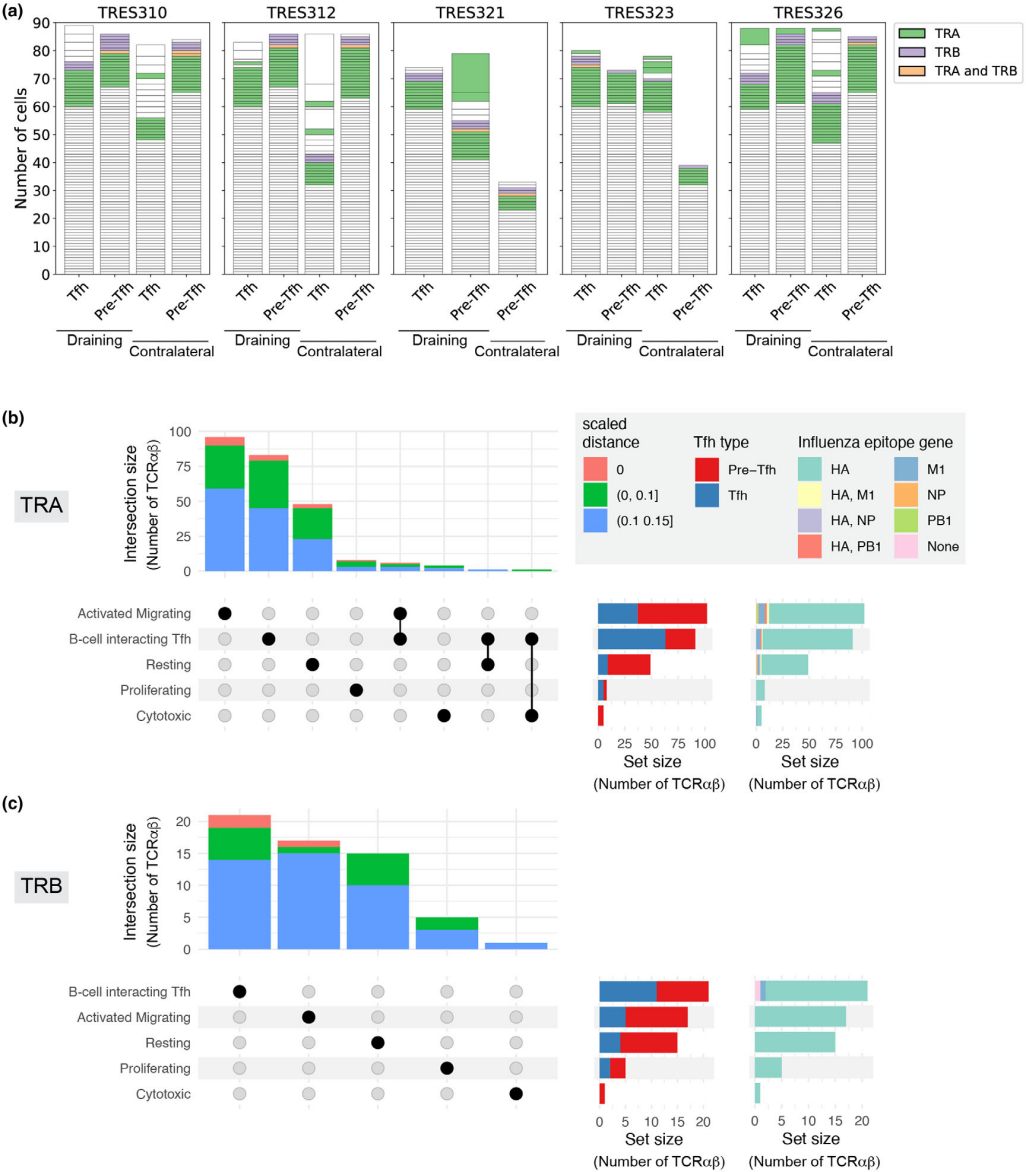
TCRɑβ observed in multiple clusters mapped to previously reported influenza‐specific T‐cell receptor (TCR) sequences. **(a)** The number of cells per sample with TCRɑβ for which the TRA and/or TRB clonotype mapped to previously reported influenza‐specific TCR sequences. Each segment of the bar plots represents a TCRɑβ and the size of the segment indicates the number of cells with that TCRɑβ. Colour segments indicate cells for which there was a match to a previously reported influenza‐specific TRA, TRB or both TRA and TRB sequence. Upset plots of lymph nodes (LN) TCRɑβ that mapped to previously reported influenza‐specific **(b)** TCRɑ or **(c)** TCRβ amino acid sequences, showing their distribution across clusters, including TCRɑβ that were observed across multiple clusters. Also shown are the numbers of these TCRɑβ per cluster that originated from the sorted T follicular helper (Tfh) or Precursor Tfh (Pre‐Tfh) populations and that mapped to TCRs of reported influenza epitope gene specificity (right‐hand bar plots). TCRs that mapped to a previously reported influenza‐specific TCR sequence had matched V and J gene usage and a length‐scaled distance between CDR3 amino acid sequences of no greater than 0.15. The intersection size represents the number of TCRɑβ within a cluster, or common across clusters, and the set size represents the number of TCRɑβ per cluster. The TCRɑβ common between the B‐cell‐interacting Tfh and either Activated Migrating or Resting clusters had identical TCRɑ and TCRβ nucleotide sequences. The TCRɑβ common between the B‐cell‐interacting Tfh and Cytotoxic clusters had identical TCRɑ nucleotide sequences and TCRβ amino acid sequences, with the CDR3β sequences differing by a single nucleotide (i.e. Clones 23 and 24 in Figure 2b).

To investigate whether Pre‐Tfh or Tfh cells responding to the influenza vaccine were among the sampled TCR repertoires, and among the cells exhibiting potential functional flexibility, we mapped the LN TCR data against previously reported influenza‐specific TCRs^39^, ^40^, ^41^, ^42^, ^43^, ^44^ (in total 6153 TRA and 9198 TRB sequences; summarised in Supplementary figure 3a). TCRs that mapped to a previously reported influenza‐specific TCR sequence were identified by matched V and J gene usage and a length‐scaled distance between CDR3 amino acid sequences of no greater than 0.15. Cells with TCRα and/or TCRβ with inferred influenza specificity were observed in all LN samples (Figure 3a) and in all five clusters, including TCRɑβ that were observed across multiple transcriptionally distinct clusters (Figure 3b and c). There was also a small subset of these TCRɑβ for which both the TCRα and TCRβ mapped to influenza‐specific TCR sequences (Figure 3a, Supplementary figure 3d). Cells with TCRα or TCRβ matches to the database of influenza‐specific TCRs were associated with both the Pre‐Tfh and Tfh cell populations (Figure 3a–c). Although previously reported influenza‐specific TCRs used in this analysis featured a diversity of influenza genes (Supplementary figure 3a), the LN‐TCR matches to the database were largely to TCRs with reported influenza specificity associated with the haemagglutinin (HA) gene for both exact and inexact matches (Figure 3b and c; Supplementary figure 3b and c). The vaccine administered in this study was the Influvac® Tetra (Mylan), which contains 15 μg of HA from four influenza strains, providing encouraging support that the cells with TCRs mapping to previously reported influenza HA‐specific TCRs may be responding to vaccination.

### Differentiation of Pre‐Tfh to Tfh is marked by functional transitions and distinct phases of activation

**Figure 4:**
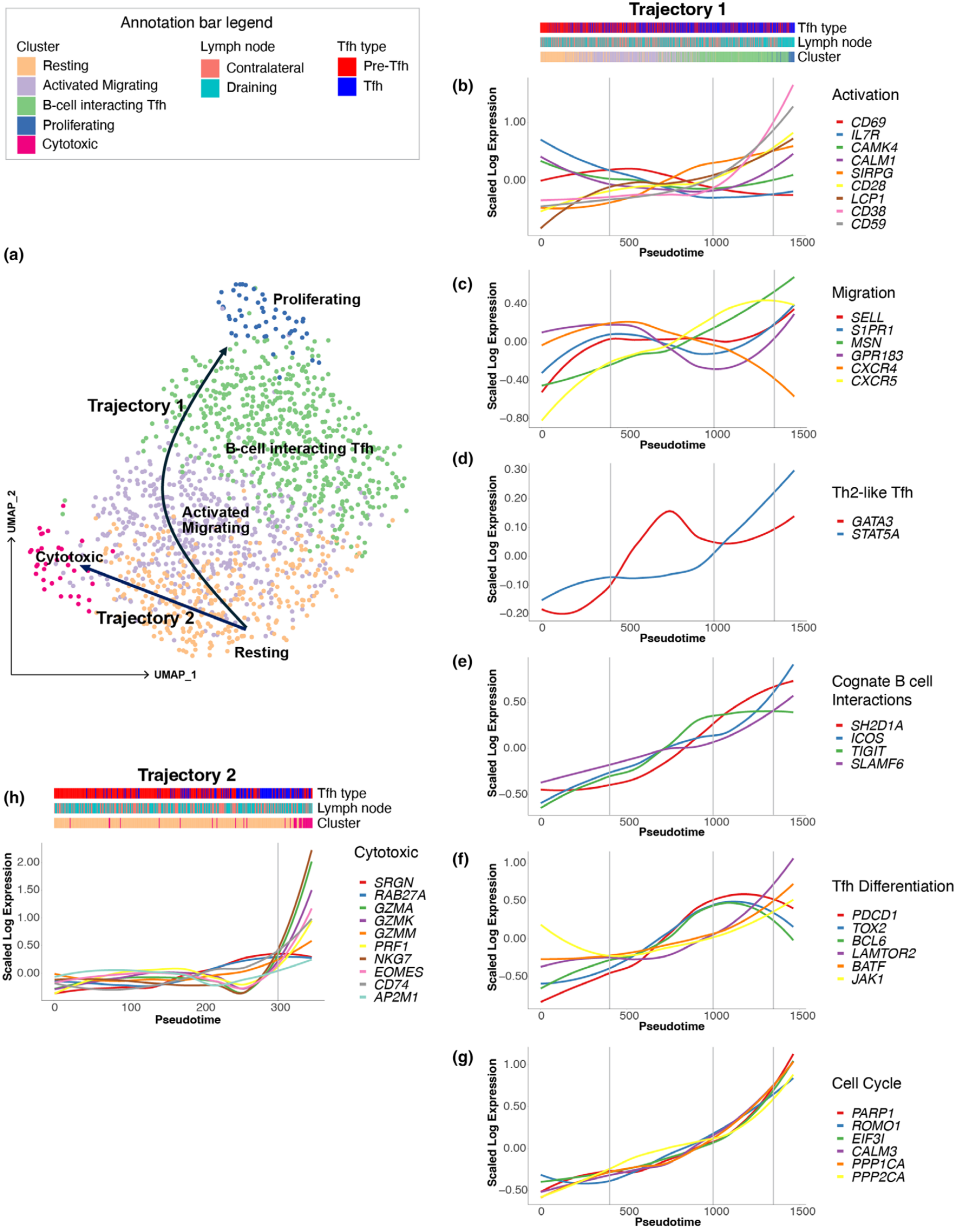
Functional transitions observed by changes in gene expression along inferred pseudotime. **(a)** Schematic of differentiation trajectory overlayed onto the cluster Uniform Manifold Approximation and Projection (UMAP). Loess plots demonstrating changes in the expression of genes associated with cellular activation state and effector function along inferred pseudotime for Trajectory 1 (Resting to Proliferating clusters) for genes associated with **(b)** activation, **(c)** migration, **(d)** Th2‐like T follicular helper (Tfh), **(e)** cognate B‐cell interactions, **(f)** Tfh differentiation and **(g)** cell cycle. Loess plot demonstrating changes in gene expression along inferred pseudotime for Trajectory 2 (Resting to Cytotoxic clusters) for cytotoxic genes **(h)**. Vertical lines in **(b–h)** indicate the start of functional transitions along inferred pseudotime.

To better understand the functional flexibility of Pre‐Tfh and Tfh cells, we investigated the possible differentiation trajectory of these cells. The traditional approach to CD4^+^ T‐cell lineage classification establishes each subset as the sole executor of a given role or function. For example, Tfh cells have typically been defined as B‐cell helpers within the GC, providing the necessary cognate interactions required to generate highly somatically mutated antibodies. However, the presence of subsets of Tfh cells with mixed CD4^+^ T‐cell lineages (Figure 1f) suggests a more complex lineage differentiation and classification model. Here, we used inferred pseudotime and trajectory analysis to delineate functional transitions and illuminate the progression through distinct phases of activation. To determine the trajectories, we first used a probabilistic graph‐based approach (PAGA) to quantify the inter‐cluster connectivity (Supplementary figure 4a), which showed connections between the Resting cluster and both Cytotoxic and Activated Migrating clusters. PAGA also suggested connectivity between the B‐cell‐interacting Tfh cluster and both the Activated Migrating and Proliferating clusters. We also observed that the Proliferating and B‐cell‐interacting Tfh clusters had a higher proportion of Tfh cells (Figure 1d), and the Proliferating cluster had a higher proportion of cells from the draining than contralateral LNs. Finally, expanded TCR clones resided predominantly in the B‐cell‐interacting Tfh cluster, suggesting that this cluster was further along the differentiation pathway (Supplementary figure 4e and f). Taken together, and after running the pseudotime Slingshot algorithm,^45^ this is suggestive of a bifurcating trajectory rooted in the Resting cluster that terminates at either the Proliferating (Trajectory 1) or Cytotoxic (Trajectory 2) clusters (Figure 4a). These two trajectories were then used to investigate the gene expression along pseudotime (Figure 4b–h), revealing the loss or gain of gene expression that can be mapped to clusters representing distinct cellular subsets.

Focusing first on Trajectory 1, the earliest timepoint was rooted in the Resting cluster formed predominantly by Pre‐Tfh cells, where markers of early T‐cell activation, including *CD69*, *IL7R, CAMK4* and *CALM1*, were initially highly expressed relative to later pseudotime values (Figure 4b). Furthermore, this early timepoint was characterised by relatively high expression of inhibitors of cellular proliferation and Tfh differentiation, such as *MALAT1*, *DUSP1* and *TXNIP* (Supplementary figure 5b), and *CXCR4*, which has been shown to determine T‐cell location in the LN (Figure 4c),^46^ as well as relatively low expression of the B‐cell follicle homing receptor *CXCR5* (Figure 4c) and Tfh cell markers *PDCD1* (PD‐1) and *BCL6* (Figure 4f). Taken together, this unique gene expression profile establishes cells at the beginning of the differentiation trajectory as a population less polarised towards the Tfh cell lineage that are located outside the B‐cell follicle.

The first functional transition, from the Resting to Activated Migrating clusters, along pseudotime was marked by the increased expression, relative to earlier pseudotime, of migratory markers that are essential to Tfh cell homing to the paracortex of the LN. The genes underpinning this transition to a new functional subpopulation include: *SELL* (CD62L),^47^ *S1PR1*, *MSN* and *GPR183* ^48^ (Figure 4c). Sustained expression of *CD69* was also observed through the first pseudotime transition (Figure 4b), but the remaining markers of early activation were decreasing in expression. The change in the activation state was preceded by sustained expression of *SIRPG* ^49^ and a marked increase in the expression of both *CD28* ^50^ and *LCP1* (Figure 4b), which play a role in activation following TCR/CD3 and CD28 costimulation and regulate surface expression of CD25 and CD69.^51^ Interestingly, a gain in Th2 effector function was observed prior to this transition in the trajectory, identified by increased expression of *GATA3* and *STAT5A* (Figure 4d). Overall, these results demonstrate a transition to a population of cells with a highly motile gene expression profile that is highly activated following TCR stimulation, suggesting that these cells are migrating through the LN paracortex surveying for and interacting with antigen.

At the second functional transition in pseudotime, we observed sustained expression of activation markers associated with TCR stimulation, accompanied by a loss of expression of early activation markers (Figure 4b) and genes inhibiting Tfh cell differentiation (Supplementary figure 5b). There was a decrease in expression of migratory genes associated with the LN paracortex (*CD44* and *CCR7*; Supplementary figure 5b and *CXCR4*; Figure 4c), accompanied by increasing expression of the B‐cell follicle homing gene *CXCR5* and a local minimum in expression of *S1PR1* and *GPR183* (Figure 4c). The localisation to the B‐cell follicle is consistent with the increased expression of key GC Tfh lineage genes (*PDCD1* (PD‐1), *TOX2* and *BCL6*; Figure 4f) and genes required for the formation of stable cognate interactions between Tfh and GC B cells (*SH2D1A*, *ICOS*, *TIGIT* and *SLAMF6*; Figure 4e). Of note, this is also where a shift in the sorted cell type occurs, with Tfh cells beginning to dominate the later stages of the trajectory (Figure 4, Tfh type in annotation bar). Interestingly, *CD69* expression decreased over these later stages in pseudotime (Figure 4b), suggesting these Tfh cells could be preparing to exit the LN and enter the periphery. Therefore, along the progression of pseudotime, there is a differentiation trajectory towards a more polarised Tfh cell subset with a partial Th1 gene expression profile (Supplementary figure 5b) that is localised to the B‐cell border of the GC. This is where these Tfh cells interact with GC B cells, ultimately forming stable conjugates.

The final transition of the differentiation trajectory was signified by the increase in cellular proliferative capacity, identified by high expression of *PARP1*,^52^, ^53^ *ROMO1* ^54^ and *EIF3I* ^55^ (Figure 4g). Complementary to increased expression of these proliferation genes, an increase in cell cycle associated genes was observed, including: *CALM3*,^56^ *PPP1CA* and *PPP2CA* ^57^, ^58^ (Figure 4g), and *CLTA* ^59^, ^60^ (Supplementary figure 5b). Furthermore, at the end of the trajectory, increased expression of genes associated with cytokine signalling (*LAMTOR2* and *JAK1*) and the transcription factor *BATF* was observed, suggesting further commitment to the Tfh lineage and potentially maintenance of a stable Tfh population (Figure 4f). In addition to the moderately increasing expression of costimulatory molecule *CD28*, the final stage of the trajectory was marked by a transition in activation state distinguished by a higher expression of *CD38* and *CD59* (Figure 4b). Interestingly, these late stage trajectory Tfh cells gained expression of *GBP2*, previously shown to exhibit antiviral activity against influenza virus^61^ (Supplementary figure 5b).

Focusing on the second trajectory, this component of the bifurcated trajectory from the Resting cluster to the Cytotoxic cluster was marked by the gain of a cytotoxic effector gene expression profile (Figure 4h, Supplementary figure 5c). Across the resting to cytotoxic transition, we observed a marked increase in the expression of key cytotoxicity markers, including genes encoding for granzymes A, K and M (*GZMA*, *GZMK* and *GZMM*); *PRF1* (perforin); *NKG7*, which plays a role in CD4^+^ T‐cell activation following infection^62^; and *EOMES*, transcription factor and inducer of cytolytic capacity in CD4^+^ T cells^63^, ^64^ and associated with memory T‐cell differentiation.^65^ Interestingly, leading up to the transition of cells towards the cytotoxic gene expression profile, there was a relatively moderate increase in the expression of *SRGN* and *RAB27A*, which was sustained through this transition. Furthermore, this transition was accompanied by decreased expression of key Tfh markers (*PDCD1, BCL6* and *CXCR5*), suggesting differentiation away from the Tfh lineage, resulting in a partial Tfh profile (Supplementary figure 5c). The continued expression of a partial Tfh gene profile and associated LN homing markers, concurrently with the cytotoxic profile, could be essential to the retention of this population within the LN. Finally, the end stage of this differentiation trajectory is marked by high expression of *CD74* (Figure 4h and Supplementary figure 5c), which plays an important role in the stabilisation of the TCR: MHC‐II: Antigen complex.^66^

### AUCell analysis of gene sets confirms transitions in functional states along inferred pseudotime

**Figure 5:**
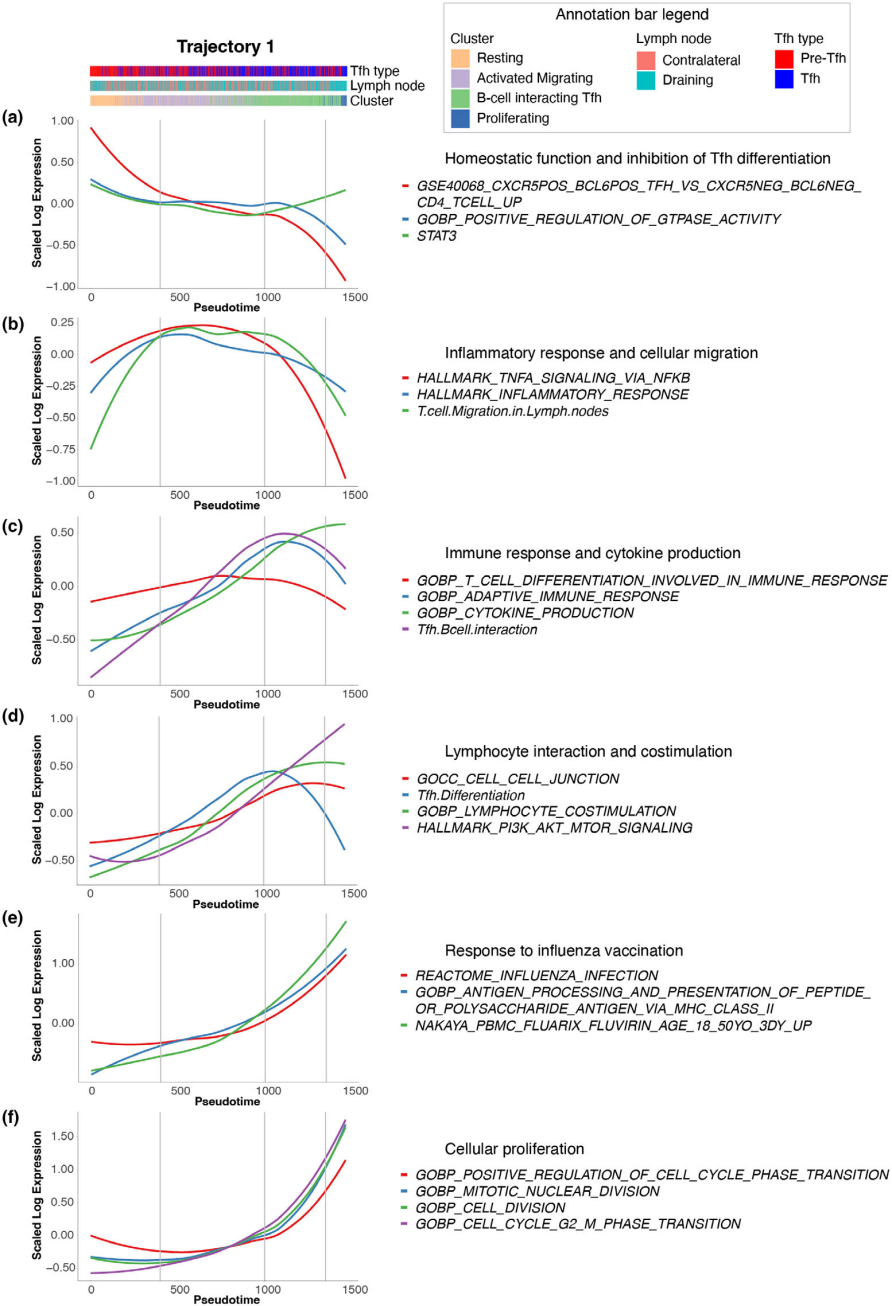
Enrichment of gene set pathways along Trajectory 1 inferred pseudotime. Loess plots demonstrating enrichment of gene set pathways along inferred pseudotime for Trajectory 1 (Resting to Proliferating clusters), depicted in Figure 4a, that are associated with **(a)** homeostatic function and inhibition of T follicular helper (Tfh) differentiation, **(b)** inflammatory response and cellular migration, **(c)** immune response and cytokine production, **(d)** lymphocyte interaction and costimulation, **(e)** response to influenza vaccination and **(f)** cellular proliferation. The expression values correspond to AUCell values calculated from the corresponding gene sets. Vertical lines indicate the start of functional transitions along inferred pseudotime.

**Figure 6:**
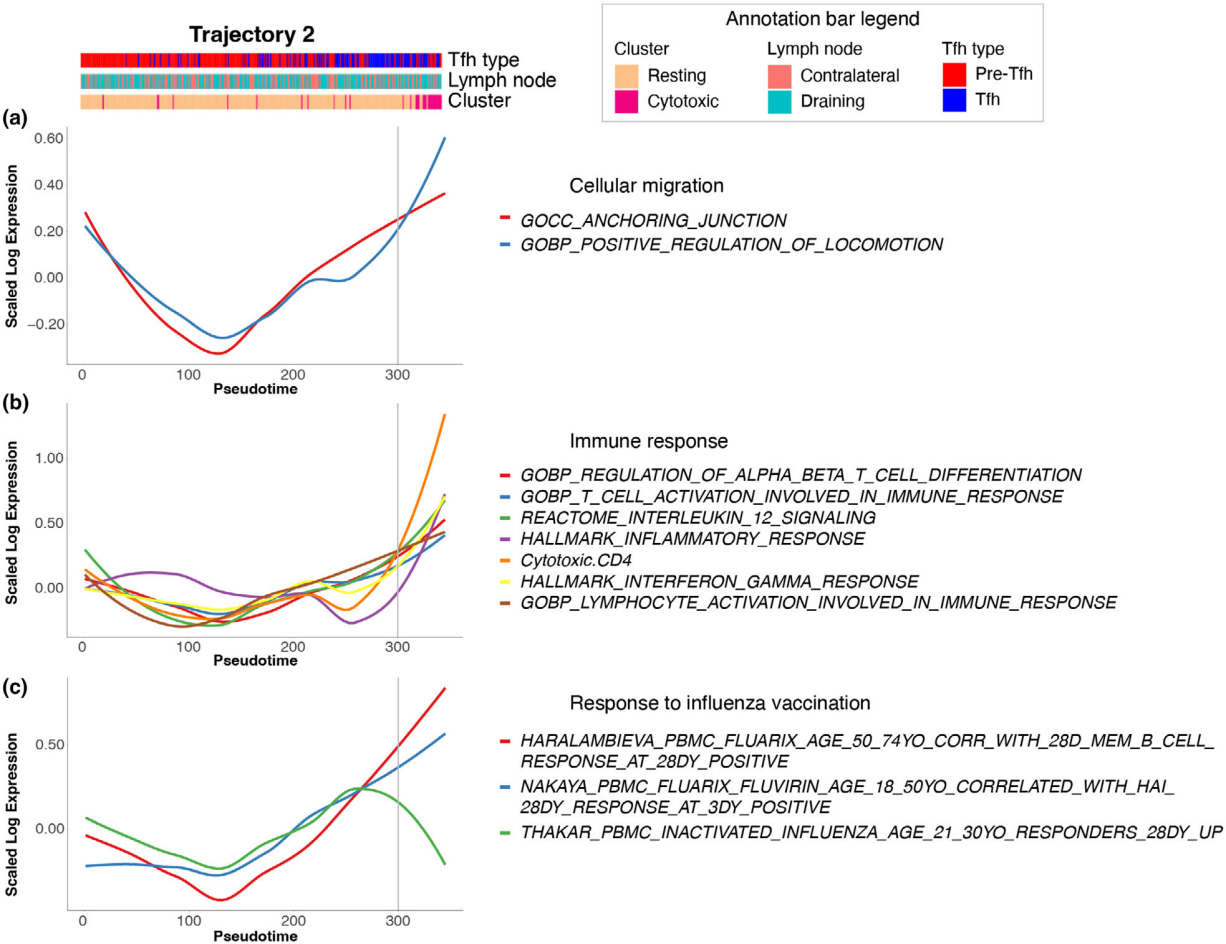
Enrichment of gene set pathways along Trajectory 2 inferred pseudotime. Loess plots demonstrating enrichment of gene set pathways along inferred pseudotime for Trajectory 2 (Resting to Cytotoxic clusters), depicted in Figure 4a, that are associated with **(a)** cellular migration, **(b)** immune response and **(c)** response to influenza vaccination. The expression values correspond to AUCell values calculated from the corresponding gene sets. Vertical lines indicate the start of functional transitions along inferred pseudotime.

Correlation coefficients were calculated for each gene pathway whereby a positive correlation with pseudotime indicated an enrichment of these genes and associated pathways along the trajectory. Loess plots summarising the AUCell analysis pathway transitions marking changes in cell states are presented in Figures 5 and 6, for Trajectory 1 (Resting to Proliferating clusters) and Trajectory 2 (Resting to Cytotoxic clusters), respectively. The corresponding heatmaps are depicted in Supplementary figure 6.

Focusing on the first trajectory, from the Resting to Proliferating clusters (Figure 4a), we observed, along the earlier stages of pseudotime, a decrease in gene pathways associated with homeostatic function and inhibition of Tfh differentiation (Figure 5a). This was accompanied by an increase in the gene pathways associated with inflammatory response and cellular migration (Figure 5b), immune response and cytokine production (Figure 5c), and lymphocyte interaction and costimulation (Figure 5d).

The later stages of the differentiation trajectory were enriched for multiple gene pathways associated with proliferation and transitions through the cell cycle phases (Figure 5f), and a sharp loss of expression of gene pathways associated with the cellular activation involved in the immune response and TNF‐α signalling (Figure 5b and c). This could be important for the maintenance of the B‐cell follicle and the GC as TNF‐α has been associated with lymphoid tissue disorganisation and GC B‐cell suppression.^67^ Of particular interest, gene pathways associated with the immune response to influenza vaccination were enriched across the entire differentiation trajectory (Figure 5e).

For the second trajectory, from Resting to Cytotoxic clusters (Figure 4a), gene pathways associated with a cytotoxic signature and IL‐12 signalling (Figure 6b), high hemagglutination inhibition (HAI) titre and memory B‐cell responses at 28 days post‐influenza vaccination (Figure 6c), and cellular migration (Figure 6a) were enriched. However, pathways indicating involvement in the immune response and cellular differentiation remained relatively stable throughout this trajectory and were not upregulated towards the end of pseudotime.

## Discussion

Considerable research on the sub‐lineages of circulating‐Tfh (c‐Tfh) cells in peripheral blood has identified Th1‐like, Th2‐like and Th17‐like c‐Tfh cells, each with varying abilities to provide help to B cells.^28^ Functional flexibility of LN Tfh cells is a promising therapeutic target for biasing the immune response towards a desired phenotype following vaccination. A recent study in mice and human tonsillar tissue demonstrated Tfh cells with diverse Th‐like phenotypes influenced B‐cell response profiles; however, a clonal relationship between Tfh phenotypes is yet to be established.^10^ Here, we leverage scRNA‐seq data to determine whether two predominantly tissue‐located cell lineages, the Pre‐Tfh and Tfh cell populations, expressed alternative or multiple lineage attributes. The expression of partial CD4^+^ T‐cell lineage profiles was observed by supervised analysis and supported by UMAP dimensionality reduction and differential gene expression analysis. Five clusters with distinct gene expression profiles, suggestive of distinct functionality, were identified based on differentially expressed genes: Resting, Activated migrating, B‐cell‐interacting Tfh, Proliferating and Cytotoxic. Using inferred pseudotime, a differentiation trajectory was constructed revealing the transition of cells through activation states and the gain and loss of different lineage attributes and effector functions. Using the cell's natural barcode, the TCR, we were able to infer divergent differentiation into different fate lineages from a common precursor cell. Furthermore, TCR sequencing analysis revealed exciting and promising initial evidence of the flexible potential within the Tfh lineage that could be leveraged to drive more efficient vaccine responses.

Pseudotime analysis identified the Resting cluster as the root of the bifurcating differentiation trajectory, marked by low lineage marker expression, early activation status and enrichment of homeostatic cellular pathways. Interestingly, this cluster exhibited high expression of *CAMK4*, previously identified as a key negative regulator in antibody responses to trivalent seasonal influenza vaccination,^68^ suggesting that these are a resting population of memory Tfh cells, poised to differentiate into more specialised antigen‐specific subsets of Tfh cells with different functions. As cells transitioned along the Resting to Proliferating trajectory from the Resting to Activated Migrating cluster, early activation markers were downregulated and TCR/CD3‐CD28 (*CD28* and *LCP1*) signalling genes were upregulated, suggesting a transition from environmental stimuli to direct TCR engagement.^51^ The LN migratory potential of this cluster was evident in expression of cellular motility genes (*SELL*, *CD44*, *CCR7*, *GPR183* and *CXCR4*), suggesting a role for migration within the LN to survey for antigen in the paracortex or providing help to B cells at the T:B‐cell border. Furthermore, activation and migratory gene sets were enriched, including signalling of the TCR complex and inflammatory response, and lymphocyte migration pathways. Whilst this cluster expressed a partial Tfh lineage gene expression profile, ensuring their retention within the LN, increasing expression of Th2 lineage transcription factor, *GATA3*, and other key Th2 genes (*STAT5A*, *IRF4* and *MAF*) and enrichment of the TNF‐α signalling via NF‐κB pathway was observed during this stage of differentiation. Th2‐like c‐Tfh cells have been shown to be better B‐cell helpers, contribute a higher proportion to the antigen‐specific c‐Tfh cell response and induce superior humoral immune responses^28^, ^69^, ^70^, ^71^, ^72^ The presence of a subpopulation of Th2‐like Tfh cells within the LN is notable, as they could improve vaccination response in immunocompromised populations.

As cells transitioned into the B‐cell‐interacting Tfh cluster, genes associated with conjugate formation and the GC reaction (*ICOS*, *TIGIT*), regulators of Tfh and B‐cell colocalisation (*CD84*,^19^, ^73^ *CD40LG*,^74^, ^75^, ^76^ *SH2D1A* ^77^, ^78^) and enrichment for pathways, such as cell‐to‐cell adhesion, lymphocyte costimulation and cytokine production were observed. Interestingly, this cluster expressed high levels of the cellular anergy gene *OTUB1*, suggesting a prolonged interaction with antigen, leading to exhaustion. This cluster of cells exhibited a partial Th1‐like gene expression profile. Although inefficient GC supporters, Th1‐like c‐Tfh cells have been shown to provide help to antigen‐specific B cells and promote the generation of high‐avidity antibodies post‐influenza vaccination.^79^, ^80^ Interestingly, influenza‐specific Th1‐like c‐Tfh cells emerge post‐vaccination^81^, ^82^ and have been reported to provide cognate B‐cell help leading to IgG2 antibody generation in mice.^83^ Th1‐like Tfh cells within the LN during a secondary immune response could play a unique role in the reactivation of pre‐existing memory B cells, ultimately allowing improved rapid recall responses to vaccination or infection.

Finally, the transition into the Proliferating cluster was marked by high expression of proliferation genes (*PARP1*, *ROMO1*, *EIF31* and *MKI67*) and cell cycle associated genes (*CALM3*, *PPP1CA*, *PPP2CA* and *CLTA*), required for Tfh differentiation, and gene set pathways, including mitotic nuclear division, positive regulation of cell cycle phase transitions and cell division. This cluster marks a transition to a gene expression profile of prolonged activation via TCR engagement and influenza immune response modulation, upregulating *CD38* ^84^ and *CD59* ^85^ respectively. Interestingly, this cluster expressed high levels of *GBP2*, shown to exhibit antiviral activity against influenza.^61^ The pro‐inflammatory phenotype of this cluster was supported by gene set enrichment of pathways, including cytokine production, antigen processing and presentation and IFN‐γ response. Together, these results suggest that, following stimulation with antigen, these Tfh polarised cells exhibit proliferative potential and antiviral activity.

A second, diverging differentiation trajectory was identified from the Resting to Cytotoxic cluster, a more terminally differentiated subset marked by high *EOMES* expression, enrichment of the IFN‐γ and inflammatory response pathways and cytotoxic capacity, along with high expression of Tfh genes indicating potential retention in the LN for antigen interaction. These cells may regulate the GC response,^53^ via killing MHC‐II expressing cells, though their role appears context dependent, being linked to improved clinical outcomes in some viral infections^86^, ^87^, ^88^, ^89^ but poorer prognosis in severe COVID‐19 infection.^90^ Overall, these results could suggest a regulatory role for Cytotoxic Tfh cells within the GC; however, further investigation into this subset is warranted.

Cellular flexibility is inherently difficult to investigate outside of highly manipulated animal models because of the need to track, on a per cell basis over time, protein and/or gene expression, transcription factor, cytokine and chemokine production. TCR sequencing provides a highly specific basis for tracking single cells of a common origin. The potential for Tfh cells to generate populations with multiple cell fates and genetic profiles has been observed in mice^91^ and post‐influenza vaccination in humans.^15^ Our single‐cell RNA‐seq data for human LN Tfh and Pre‐Tfh cells following influenza vaccination revealed multiple examples of cells with different cell lineages expressing the same TCRαβ, identical at the level of the nucleotide sequence. Perhaps the strongest evidence of cellular flexibility was observed in TCR Clone 1 that spanned four clusters with distinct inferred functionality and three T‐cell lineage gene expression profiles, namely Th2‐like, Th1‐like and Tfh. Two interesting clones observed were TCR Clones 23 and 24, identified in the B‐cell‐interacting Tfh and Cytotoxic clusters. These two clones had identical TCRα, non‐germline CDR3α and CDR3β sequences, and CDR3β nucleotide sequences that differed by one nucleotide which encoded the same amino acid sequence. As the generation probability of these two paired TCRαβ is expected to be small, the single nucleotide difference could be attributed to sequencing error; therefore, these two T‐cell clones may represent a divergent differentiation from a common precursor into two distinct T‐cell lineages, one cytotoxic and one Th1‐like Tfh lineage, *in vivo* in humans. If the single nucleotide difference is biologically accurate, this is interesting evidence of convergence whereby two cells have produced the same functional TCR through different routes. Both hypotheses warrant further investigation. Finally, TCRα and TCRβ from LN cells in this dataset, including cells observed in multiple transcriptionally distinct clusters, mapped to previously reported influenza‐specific TCR sequences and were predominantly reported as specific for the HA‐epitope,^40^ concordant with the influenza vaccination administered in this study, providing encouraging evidence that these may be influenza‐specific T‐cell clones. These are encouraging preliminary results into the potential flexibility exhibited in LN Tfh cells and the functional heterogeneity possible within an antigen‐specific T‐cell repertoire.

Overall, this work provides exciting new insights into the dynamics of the Pre‐Tfh and Tfh cell populations in human LNs following vaccination. However, the findings of this study could be further investigated with a larger sample size and the inclusion of a naïve population for both biopsied LNs to provide a clearer resting or homeostatic population and to establish a transcriptional baseline prior to vaccination. However, we hypothesise that the Tfh differentiation pathway, like other T‐cell subsets, may incorporate a cyclic component whereby pools of memory Tfh cells are established and are rapidly reactivated upon antigen stimulation or activation. Finally, most publicly available antigen‐specific TCR sequence data are reported as unpaired TCR α‐ and β‐chain sequences and lack MHC and HLA information. The absence of this additional information limits the ability to identify and confirm influenza‐specific T‐cell clones from public databases.

A growing area of vaccine research is focussed on the possibility of guiding the immune response towards a desired CD4^+^ T‐cell lineage or effector function, for example through the addition of specific adjuvants. Understanding the innate effector heterogeneity and unique adaptability and flexibility inherent in the Tfh cell population within LNs would provide novel insight into how to influence the immune response to vaccination. The findings presented here demonstrate the expression of other Th lineage profiles, which underscores the heterogeneity in the Tfh population within human LNs *in vivo*, revealing a transcriptional continuum that exists and establishing an effector gradient throughout Tfh cell differentiation. Furthermore, the presence of cells with TCRs that mapped to previously identified influenza‐specific CD4^+^ TCR sequences and that have divergent gene expression profiles is an encouraging insight that warrants further investigation and could be an interesting target in rational vaccine design and important to our understanding of immune regulation within lymph nodes.

## Methods

### Study participants, sample collection and cell sorting

Participants were healthy volunteers (*n* = 5) with a previous history of influenza vaccination, influenza infection or both from our previously described study.^34^ Demographic and experimental details for each participant are displayed in Supplementary table 1. Written informed consent was provided prior to study commencement. St Vincent's Hospital Sydney Human Research Ethics Committee (HREC/17/SVH/20; 2019/ETH03178) approved the study.

Ultrasound‐guided FNB [referred to as fine needle aspiration (FNA) by other studies^14^, ^15^, ^16^, ^72^] was performed on human axillary LNs 5 days following seasonal influenza vaccination and was processed as previously described.^34^ Briefly, LN cells in culture media [Roswell Park Memorial Institute 1640 medium +10% heat inactivated, sterile filtered Fetal Calf Serum (Gibco, Life Technologies)] were washed by centrifugation at 335× *g* for 7 min at 25°C, supernatant was discarded, and LN cells were carefully resuspended in residual media. Cells allocated for single‐cell index Fluorescence‐activated cell sorting (FACS) were transferred to a 5 mL flow tube and 2 × the manufacturers' suggested test volumes of fluorescently labelled monoclonal antibodies [CD3 PERCP‐Cy5.5 (clone SK7 (Leu‐4))], CD4 BV605 (clone RPPA‐T4), CD20 APC‐Cy7 (clone L27) and PD‐1 BV421 (clone EH12.1) (BD Biosciences); CD45RA AF700 (clone HI100) (BD Pharmingen); and CXCR5 PE/Dazzle594 (clone J252D4) (BioLegend) were added to each sample (Supplementary table 2) and incubated for 15 min in the dark at room temperature. Cells were washed with 3 mL of Dulbecco's Phosphate Buffered Saline by centrifugation at 335× *g* for 7 min at 25°C. Supernatant was carefully discarded, and cells were resuspended in 500 μL of culture media and filtered through a 70 μm filter cap prior to sorting and stored at 4°C. Following single colour compensation, single cells were index sorted on a BD FACS Aria Fusion or BD FACS Aria III with a 100 μm nozzle at a sheath pressure of 20 psi into a 96‐well PCR plate containing cell lysis buffer reagents for plate‐based scRNA‐seq: [Triton X‐100 solution, 0.2% (0.95 μL) and Oligo‐dT primer (5 mm) 5′‐AAGCAGTGGTATCAACGCAGAGTACT30VN‐3′ (0.5 μL) (Sigma‐Aldrich); RNase Inhibitor (0.05 μL; Scientifix Pty Ltd); dNTP mix (10 mm) (0.5 μL; Promega Corporation)]. GC Tfh cells were defined for FACS as CD3^+^CD4^+^CD45RA^−^PD‐1^high^CXCR5^high^ and Pre‐Tfh cells were defined for FACS as CD3^+^CD4^+^CD45RA^−^PD‐1^+^CXCR5^+^ (Supplementary figure 1). Immediately after sorting, plates were sealed and centrifuged at 335× *g* for 15 s and stored at −80°C.

### Generation of sequencing libraries

Sequencing libraries were generated using the Smart‐Seq2 protocol.^92^ Once preparation of all sequencing libraries was complete, library pools were purified using AMPure Beads at a 0.7× ratio, followed by a second purification at 0.65× ratio and sequenced on Illumina Nova‐Seq platform. Library pools from an additional two participants were purified using AMPure Beads at a 0.6× ratio and sequenced on Illumina Next‐Seq platform.

### Alignment of single‐cell RNA‐seq reads

Post sequencing, demultiplexing was performed by the Ramaciotti Centre for Genomics, and data were delivered in FASTQ format. Raw FASTQ read quality was assessed and reads were trimmed and filtered using the Trimmomatic software (version 0.39^93^). Using the parameter, *MINLEN =* 50, reads below this length were removed. The start and end of each read were trimmed using *LEADING =* 3, *TRAILING =* 3 parameters and *SLIDINGWINDOW =* 4:10. Reads that passed quality control were aligned to the GRCh38 reference genome using STAR (version 2.7.3a^94^) with default parameters. Ensemble transcript IDs were converted to Ensembl gene IDs using the Ensembl BioMart database. Gene expression was quantified using RSEM (version 1.3.1^95^), and expression counts were normalised using SAVER with default values.^96^ Prior to downstream analysis, Ensembl gene IDs were converted to HUGO Gene Nomenclature Committee symbols.

### Extraction of full‐length TCR sequences

Reconstruction of full‐length TCR sequences from demultiplexed, raw FASTQ files was performed using VDJPuzzle (version 2^97^). Reads were quality scored, trimmed and aligned to the human reference genome GRCh38 using Trimmomatic, as above. TopHat2 was used for *de novo* assembly and error correction of reconstructed TCR contigs, which were subsequently compared with the IgBlast database. Output consisted of V, D and J gene usage, full‐length nucleotide, and amino acid TRA and TRB sequences. Filtering unproductive sequences (such as sequences that contain a stop codon) was performed within the VDJPuzzle pipeline.

## Data analysis

Data analysis followed a similar pipeline to our previous work^98^, ^99^ and was performed using the statistical programming language R (version 4.1.2^100^) within R Studio (version 2022.02.1) and Python (version 3.7^101^) within Spyder (version 5.0.5^102^). Unless otherwise indicated, a combination of custom scripts and functions from the R packages *Seurat* (version 4.0^103^) and *SingleCellExperiment* (*SCE*, version 1.16^104^), were used for data mining, wrangling and analysis; a combination of functions from the base R plotting library and ggplot2 R library (version 3.3.5^105^) were used to generate figures. Samples were quality controlled to remove cells with low mitochondrial content and low/high numbers of unique genes and total counts using the QC thresholds: minimum total counts = 8000, minimum unique genes = 450 (for TRES323 and TRES326 samples) or 800 (for TRES310, TRES312 and TRES321 samples), maximum unique and total genes = 0.999 quantile, and maximum mitochondrial ratio = 0.4. Gene expression was normalised using *scran* while protein expression was normalised using centred log ratio (CLR) normalisation. Samples were integrated using Seurat's *FindIntegrationAnchors* and *IntegrateData* functions (version 4.0^103^). Clusters were determined by Seurat's *FindNeighbours* and *FindClusters* functions. A range of resolution parameters (0.01–6) was initially tested to determine, which values produced biologically meaningful clusters. This assessment was based on differential gene expression analysis at each resolution and by examining gene expression patterns across clusters on the UMAP. Based on these evaluations, a final resolution of 0.55 was selected.

The package *AUCell* ^106^ was used to assign gene signature expression scores. Pathways and gene sets were downloaded from the MsigDB^107^ in combination with the curated T‐cell specific gene set (Supplementary table 6). To obtain the pathways, we first ran GSEA per cluster for each batch and then calculated the normalised enrichment score (NES). Manually selected pathways were then chosen if the NES > 1.3 in at least one cluster. The DGE analysis was performed using Seurat's *FindAllMarkers* function with the ‘MAST’ option. Default parameters for all functions were used unless indicated in the methods. The minimal number of nucleotide additions required in the generation of the TRA and TRB sequences was determined from gene alignments of the full‐length TCR sequences by IMGT/HighV‐QUEST.^108^ TRA and TRB sequences for the LN cells were mapped to previously reported TCR sequences of influenza‐specific CD4^+^ T cells (VDJDb,^39^ downloaded 30 July 2024, and published data^40^, ^41^, ^42^, ^43^, ^44^), with a successful match requiring matched V and J gene usage and a length‐scaled Levenshtein distance between CDR3 amino acid sequences of no greater than 0.15.

### Trajectory analysis

A differentiation trajectory was constructed using a combination of three computational tools and validated by TCR repertoire analysis, *a priori* gene expression and protein median fluorescence intensity (MFI) analysis (summarised in Supplementary figure 4).

Partition‐based graph abstraction (PAGA) quantifies transitions in biological state by estimating the connectivity of clusters, represented as nodes, and constructing a graph‐like map for easy interpretation.^109^ The PAGA plots were generated using the *scanpy* package (version 1.8.1^110^), by first performing dimension reduction using the function *pca*, finding the nearest neighbours using the function *neighbours*, and finally running the function *paga* using default parameters on the integrated gene expression matrix and using the pre‐defined clusters.

Identifying the differences and similarities between closely related subsets of cells, such as Tfh and Pre‐Tfh cells, is reliant on the construction of a smooth transition between cellular states. Here, we used Slingshot to infer cell lineages and pseudotime from the single‐cell gene expression data. Slingshot was applied to the UMAP formed from the integrated gene expression matrices using the *getLineages* and *getCurves* functions in the *slingshot* package (version 2.20^45^), and by manually assigning an initial root value. The pseudotime values generated from Slingshot were used to generate the smoothed gene expression and AUcell vs scaled pseudotime curves, where the smoothed expressions were calculated using the *geom_smooth()* R function with default parameters and the pseudotime was scaled to take on values between 0 and 1.

Although PAGA identifies the connections between clusters, it offers no information about the direction of the differentiation relationship between connected populations. This can be achieved by performing RNA velocity analysis whereby cellular differentiation states and lineage decisions are constructed using inferred gene‐specific transcription, splicing and degradation rates.^111^ RNA velocity was implemented by first extracting the unspliced and spliced matrices generated from the Smart‐SeqII data using *bustools*, and then running the standard RNA velocity pipeline (*scvelo*: *filter_and*_*normalize*, *moments*, *recover*_*dynamics*, *velocity*) using default parameters and the ‘dynamical’ mode.^111^ The final RNA velocity plot was generated by concatenating the individual RNA velocity plots.

The package *Cellrank* can combine the connections between clusters provided by PAGA and the directionality information provided by RNA velocity, aiming to improve the accuracy of trajectory predictions and reveal intermediate cell states.^112^ The *Cellrank* python package (version 1.5.0^112^) was used to calculate the cluster transition probabilities using the *transition_matrix* function on each sample, with the result obtained by averaging across the samples.

## Author contributions

**Hannah Law:** Formal analysis; investigation; methodology; writing – original draft; writing – review and editing. **Raymond H. Y. Louie:** Formal analysis; methodology; writing – original draft; writing – review and editing. **Omid R. Faridani:** Methodology. **Alexandra Carey Hoppé:** Methodology. **Mollie Boyd:** Writing – review and editing. **Mengfei Chen:** Writing – review and editing. **Jerome Samir:** Methodology. **Solange Obeid:** Methodology. **Brad Milner:** Methodology. **Fabio Luciani:** Writing – review and editing. **Anthony D. Kelleher:** Conceptualization; funding acquisition; investigation; supervision; writing – review and editing. **Vanessa Venturi:** Methodology; supervision; visualization; writing – review and editing. **C. Mee Ling Munier:** Conceptualization; funding acquisition; investigation; methodology; project administration; supervision; writing – review and editing.

## Conflict of interest

The authors declare no conflicts of interest.

## Supporting information


Supplementary appendix 1

## Data Availability

The data supporting the findings of this study are available from the corresponding authors upon reasonable request.
