# Supplementary material for: Human axillary lymph node T follicular helper (Tfh) and Precursor‐Tfh cells exhibit functional flexibility following seasonal influenza vaccination
Source: Clin Transl Immunology. 2025 Oct 23;14(10):e70056. doi: 10.1002/cti2.70056 (PMC12550272; doi:10.1002/cti2.70056)
Supplement: Supplementary file 1 — Supplementary appendix 1 [file CTI2-14-e70056-s001.zip › cti270056-sup-0001-Supinfo1.docx]

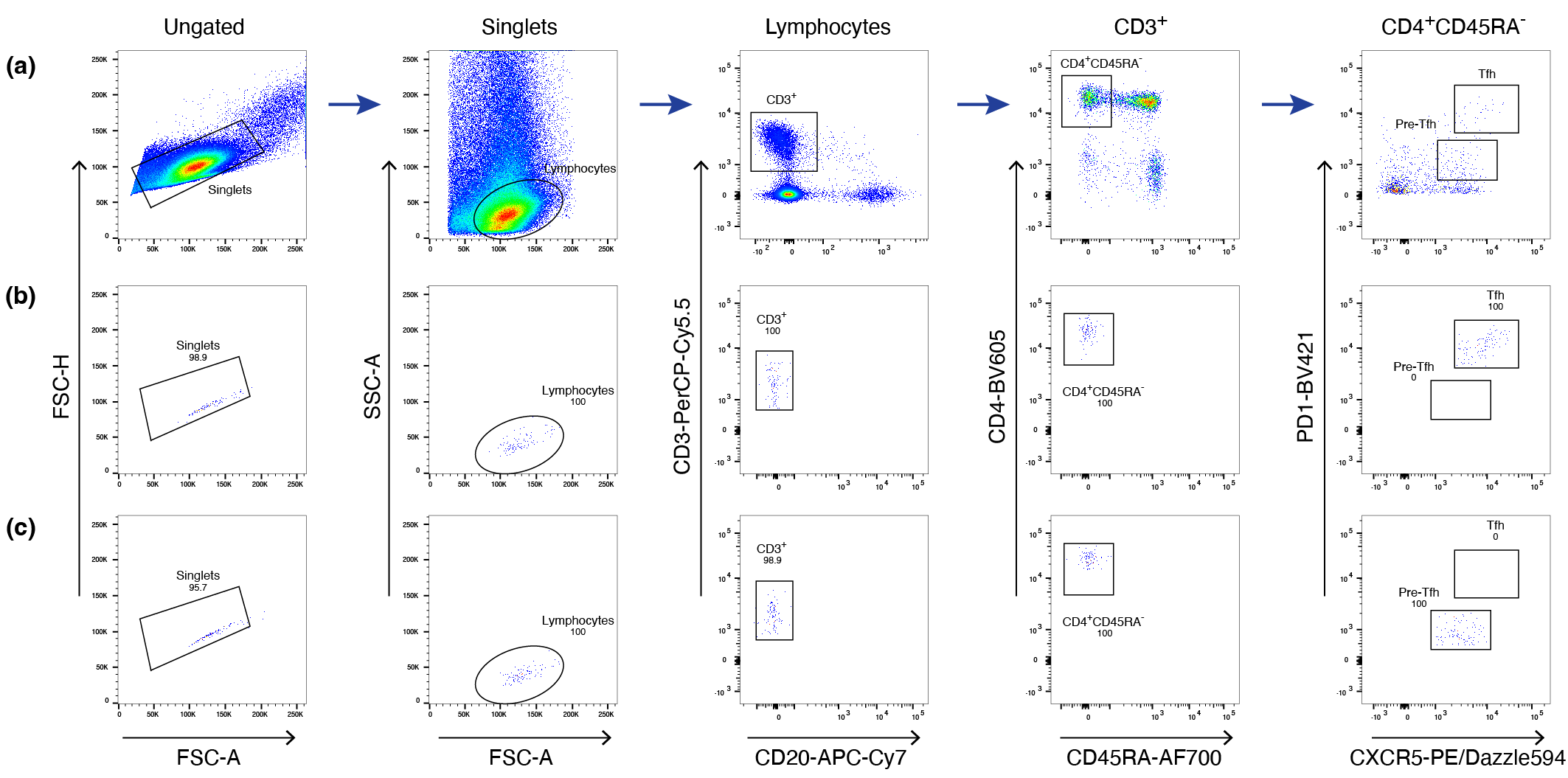


**Supplementary figure 1: *Representative flow plots for the index sorting of the Tfh and Pre-Tfh cell populations***

Representative flow plots for (a) the index sorting from bulk cells of the Tfh and Pre-Tfh cell populations, FSC-H vs FSC-A used to gate single cells, then SSC-A vs FSC-A used to identify lymphocytes, CD3 vs CD20 used to identify CD3^+^ T cells, then CD4 vs CD45RA to identify memory CD4^+^ T cells and finally, PD-1 vs CXCR5 to identify Tfh (CD3^+^CD4^+^CD45RA^-^PD-1^high^CXCR5^high^) and Pre-Tfh (CD3^+^CD4^+^CD45RA^-^PD-1^+^CXCR5^+^) cells. Representative flow plots of the index sorted (b) Tfh cell population and (c) Pre-Tfh cell population. Panels (b) and (c) provide evidence of the quality of the gating strategy for the discrimination of the Tfh and Pre-Tfh cell populations.


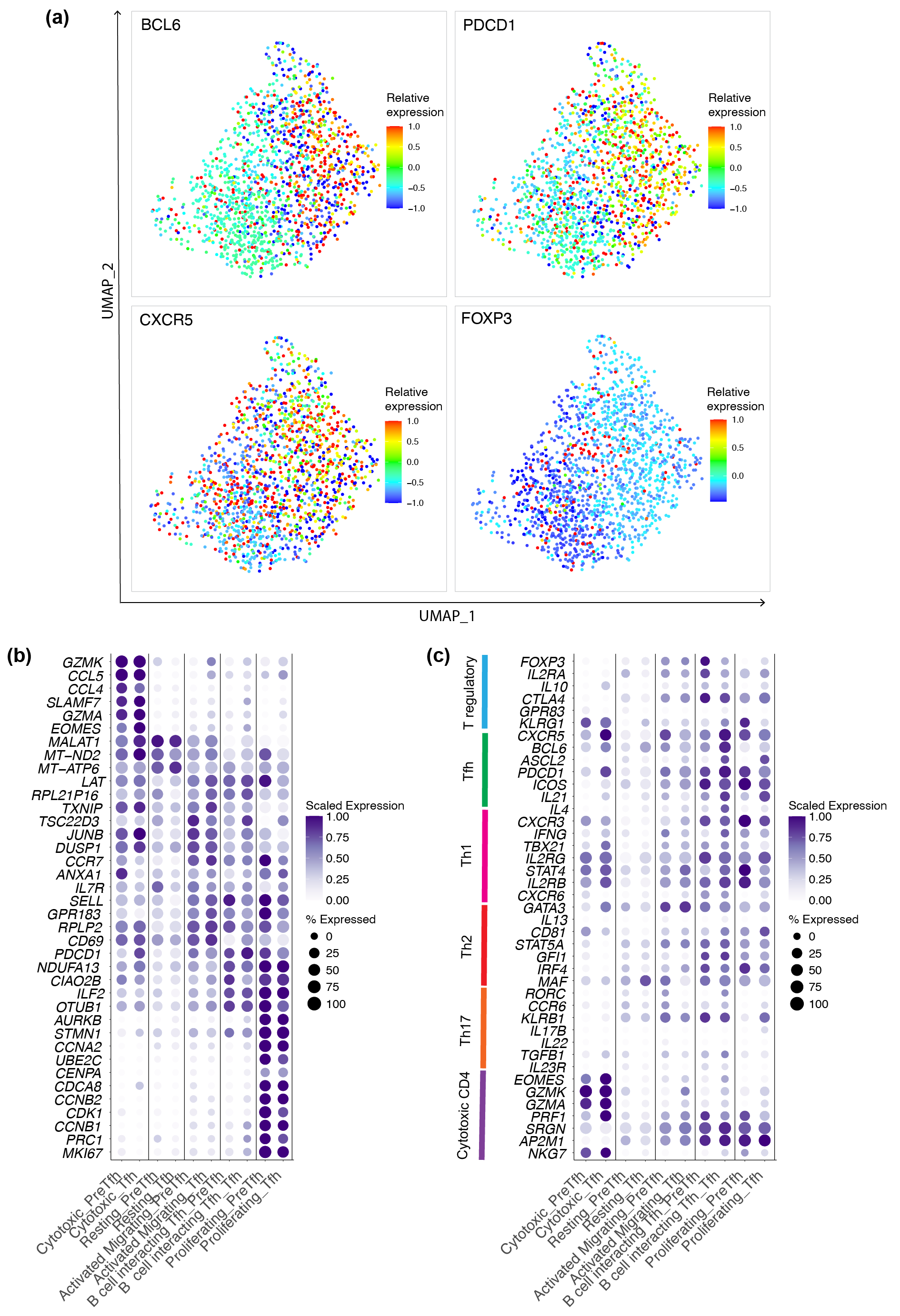


**Supplementary figure 2: *Gene expression profiles of Tfh and Pre-Tfh cells.***

(a) UMAP with cells coloured by relative expression level, truncated between 1 and -1, of the BCL6, PDCD1, CXCR5 and FOXP3 genes. Gene expression profiles per cluster for the sorted Pre-Tfh and Tfh cell populations for (b) cluster-defining genes and (c) an *a priori* gene list for the major CD4^+^ T cell lineages. Size of the dots represents percentage of cells within a cluster expressing a given gene and colour represents scaled expression values.


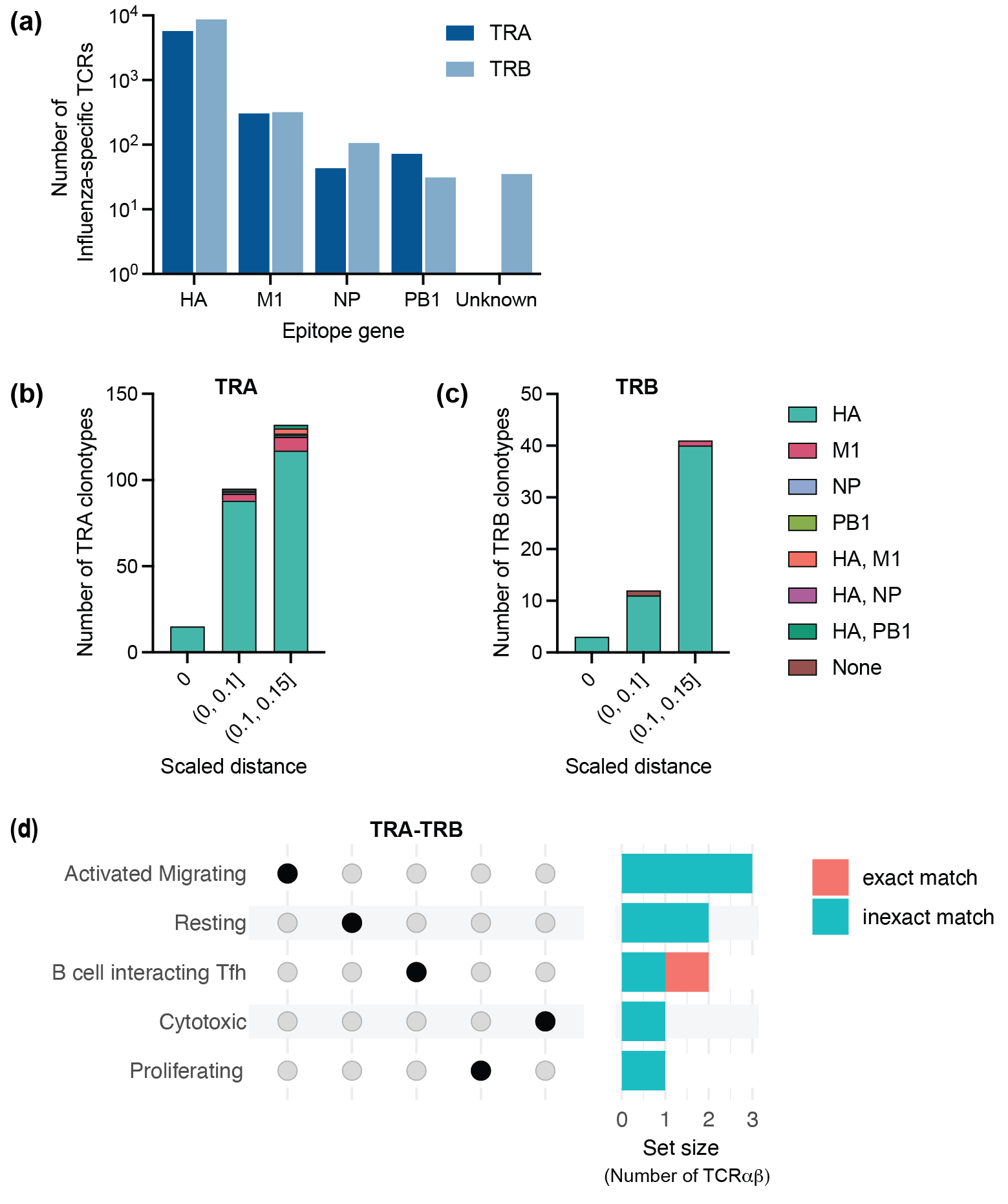


**Supplementary figure 3: *Mapping of LN TCRɑ and TCRβ sequences to previously-reported influenza-specific TCR sequences***

(a) The epitope genes associated with previously-reported influenza-specific TCRɑ and TCRβ sequences against which the LN TCR sequences were mapped. TCRs that mapped to a previously-reported influenza-specific TCR sequence had matched V and J gene usage and a length-scaled distance between CDR3 amino acid sequences of no greater than 0.15. The epitope genes of the previously-reported influenza-specific (b) TCRɑ or (c) TCRβ sequences that the LN TCR sequences mapped to, for three categories of scaled distance between CDR3 amino acid sequences: 0 (an exact match), 0<scaled distance≤0.1 (0, 0.1], and 0.1<scaled distance≤0.15 (0.1, 0.15]. (d) Upset plot of LN TCRɑβ for which both the TCRɑ and TCRβ mapped to previously-reported influenza-specific TCR sequences, showing their distribution across clusters and the number of TCRɑβ per cluster (i.e. set size in RHS bar plot) for which both the TCRɑ and TCRβ had an exact match or inexact match (i.e. 0<scaled distance≤0.15). There was one TCRɑβ in the B cell interacting Tfh cluster with an exact match to previously-reported influenza-specific TCRɑ and TCRβ sequences.

*
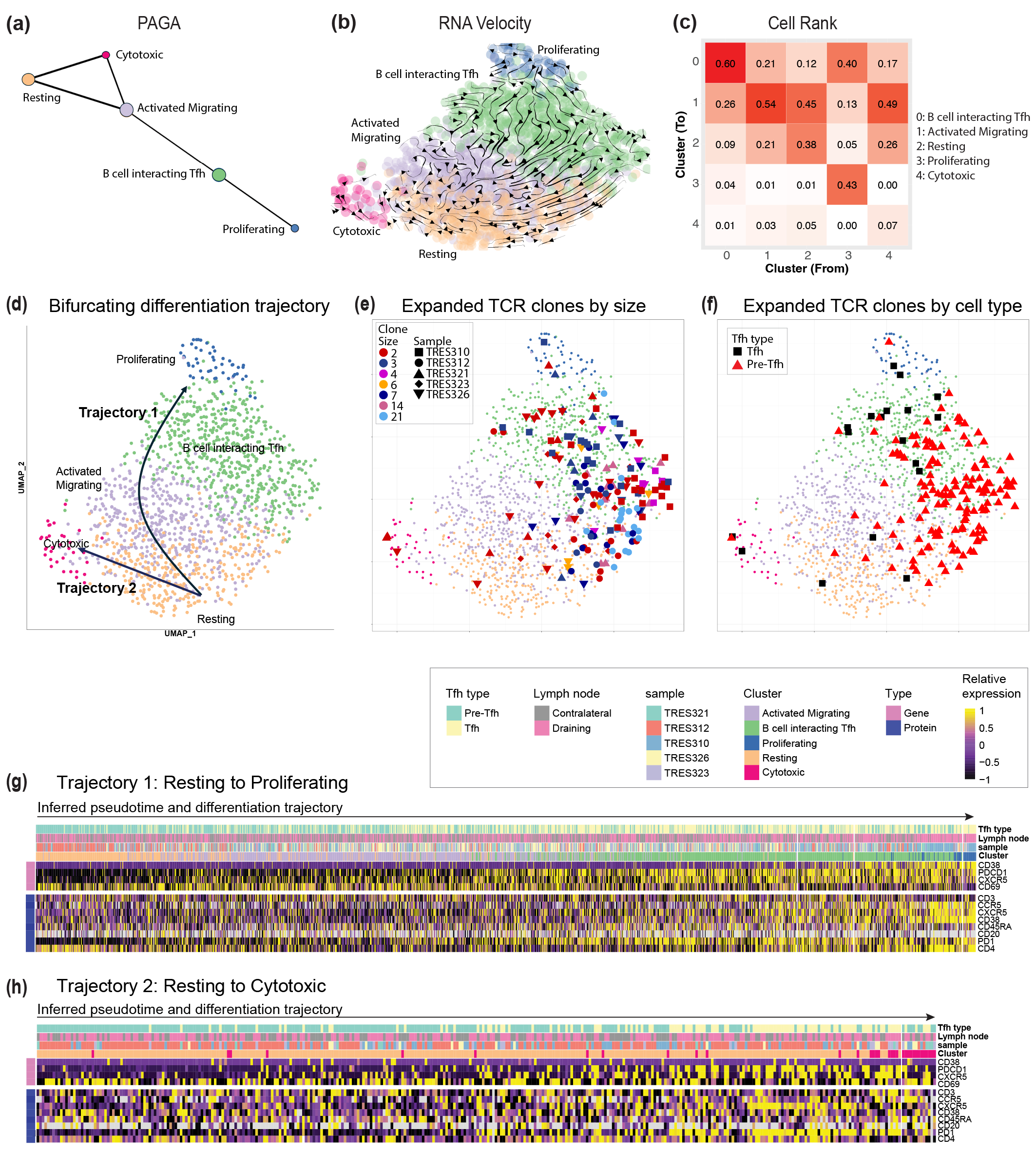
*

**Supplementary figure 4: *Evidence for the construction of the differentiation trajectory***

(a) Partition-based graph abstraction (PAGA) map revealing the connection between key clusters. (b) RNA velocity plot of the integrated UMAP data indicating the Resting cluster could be a potential trajectory root. RNA velocity supported possible differentiation from Activated migrating to B cell interacting Tfh and subsequent Proliferating clusters. However, cycling is observed between these clusters and indicates a highly complex differentiation trajectory. (c) CellRank cluster transition probability calculations support a trajectory from the Resting to Activated migrating cluster (Cell rank=0.45) and further from the Activated migrating to B cell interacting Tfh cluster (Cell rank=0.21). (d) Proposed bifurcating differentiation trajectory consisting of two diverging trajectories rooted in the Resting cluster, one terminating in the Proliferating cluster and the other in the Cytotoxic cluster. Expanded TCRɑβ clones projected onto the UMAP coloured by (e) clone size or (f) Tfh cell type. Protein MFI and selected gene expression along inferred pseudotime of (g) Trajectory 1 (Resting to Proliferating clusters) and (h) Trajectory 2 (Resting to Cytotoxic clusters).

**
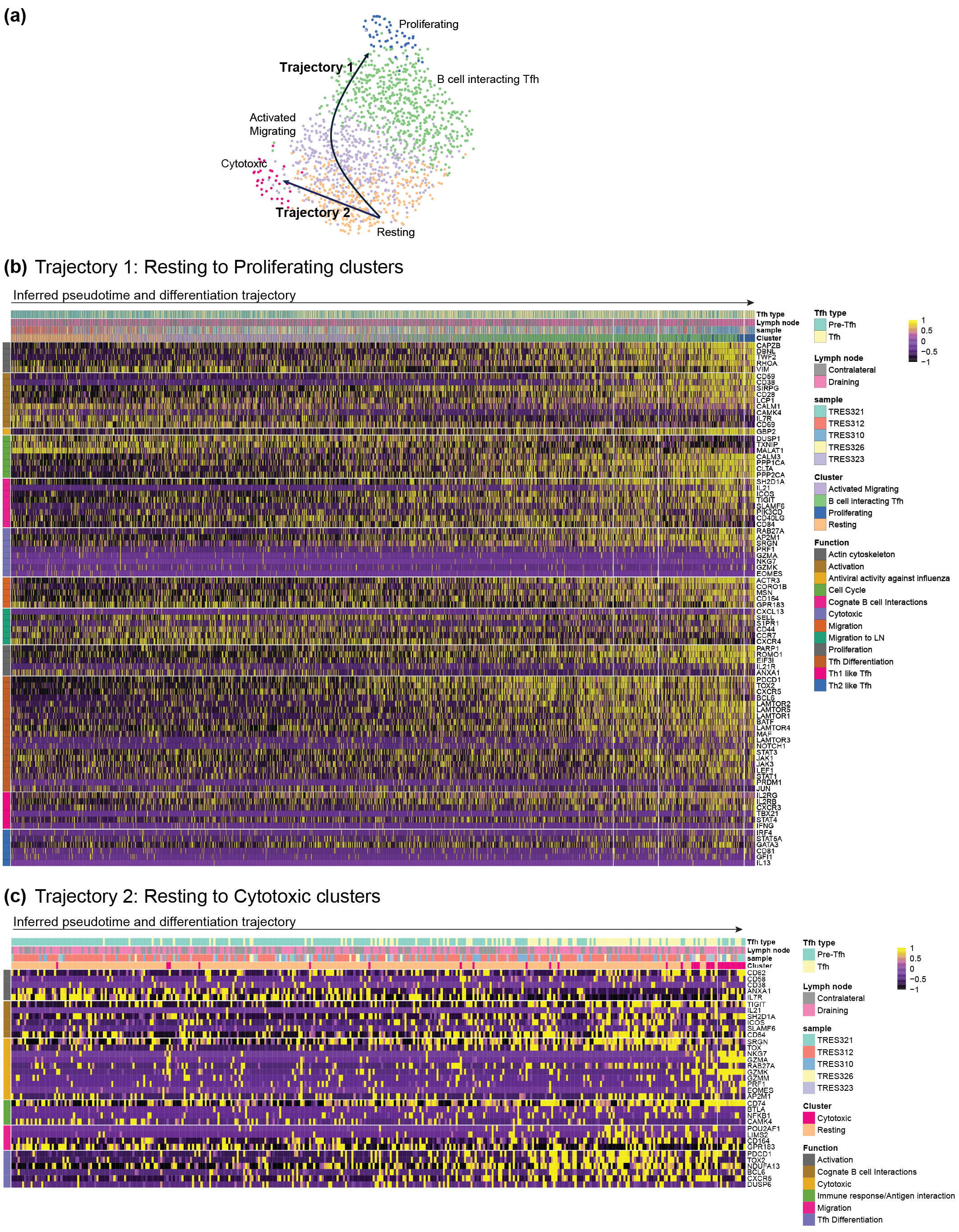
**

**Supplementary figure 5: *Transitions in activation state and effector function along inferred pseudotime***

(a) UMAP demonstrating the bifurcating differentiation trajectory through the 5 distinct cell clusters. (b) Gene expression across inferred pseudotime and along differentiation Trajectory 1 (Resting to Proliferating clusters). At the beginning of pseudotime, genes identified as early markers of activation and inhibitors of the Tfh cell differentiation program were highly expressed. This transitioned to direct cellular activation through the TCR/CD3 and CD28 costimulation complex, and ultimately to prolonged or persistent activation at the end of pseudotime. (c) Gene expression across inferred pseudotime and along differentiation Trajectory 2 (Resting to Cytotoxic clusters). Along pseudotime, cells increased expression of Tfh associated markers, which then decreased as cells gained a cytotoxic genotype. The final stage of this trajectory is marked by high expression of CD74, suggesting these cells are encountering antigen.


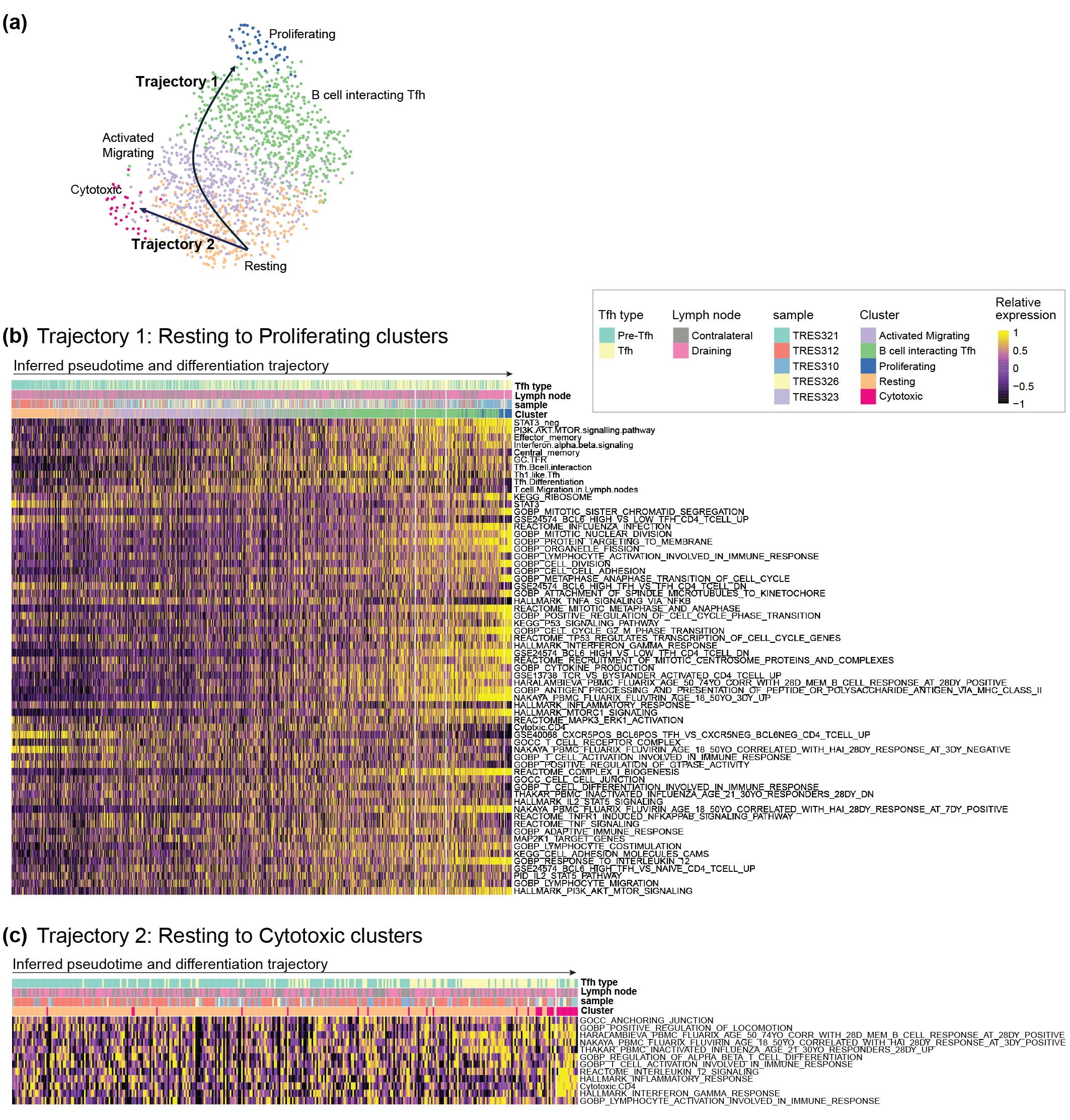


**Supplementary figure 6: *Gene pathways enriched along inferred pseudotime***

(a) UMAP demonstrating the bifurcating differentiation trajectory through the 5 distinct cell clusters. Enrichment of gene pathways from the Molecular Signature Database (MSigDB) were assessed across inferred pseudotime revealing distinct phases of the immune response to vaccination. (b) For Trajectory 1 (Resting to Proliferating clusters), the beginning of pseudotime was enriched for gene pathways associated with homeostasis and cellular survival. The first transition was marked by the gain of Th2 genotype and enrichment of TNF-ɑ signalling pathways, inflammatory response, and lymphocyte migration pathways. The second transition was characterised by a Th1-like genotype and the formation of Tfh:B cell conjugates and enriched for pathways including cell:cell adhesion, lymphocyte costimulation and T cell differentiation involved in the immune response. The final stage of inferred pseudotime involved the transition to a more polarised Tfh genotype and the enrichment for pathways including IFN-γ response, antigen processing and presentation, cell division and PI3K-AKT-MTOR signalling, essential to Tfh differentiation. (c) For Trajectory 2, during the transition from Resting to Cytotoxic clusters, there was an enrichment for gene pathways involved in cellular migration and immune response (including inflammatory response and IFN-γ response). Interestingly, gene pathways associated with cellular differentiation were not enriched, suggesting these processes were not upregulated.

**Supplementary table 1: *TRESAX study cohort details***

|  | **Participant** | | | | |
| --- | --- | --- | --- | --- | --- |
|  | **TRES310** | **TRES312** | **TRES321*** | **TRES323** | **TRES326** |
| **Age** | 46 | 33 | 55 | 64 | 26 |
| **Sex** | Female | Male | Male | Male | Female |
| **Vaccine administered (details listed in** [**^34^**](#_ENREF_34)**)** | 2019 season Influvac Tetra (influenza virus haemagglutinin; Mylan) | 2019 season Influvac Tetra (influenza virus haemagglutinin; Mylan) | 2020 season Influvac Tetra (influenza virus haemagglutinin; Mylan) | 2020 season Influvac Tetra (influenza virus haemagglutinin; Mylan) | 2020 season Influvac Tetra (influenza virus haemagglutinin; Mylan) |
| **History of influenza vaccination** | Yes | Yes | Yes | Yes | Yes |
| **Timepoint** | Day 5 post vaccination | Day 5 post vaccination | Day 5 post vaccination | Day 5 post vaccination | Day 5 post vaccination |
| **Sorted cell populations sequenced** | Draining Tfh, Draining Pre-Tfh, Contralateral Tfh, Contralateral Pre-Tfh | Draining Tfh, Draining Pre-Tfh, Contralateral Tfh, Contralateral Pre-Tfh | Draining Tfh, Draining Pre-Tfh, Contralateral Pre-Tfh | Draining Tfh, Draining Pre-Tfh, Contralateral Tfh, Contralateral Pre-Tfh | Draining Tfh, Draining Pre-Tfh, Contralateral Tfh, Contralateral Pre-Tfh |
| **Number of cells sequenced** | 368 | 368 | 239 | 368 | 368 |

* Issue with FNB sampling of the contralateral LN resulted in an insufficient number of cells for sequencing the Tfh sample and a reduced number of cells for the Pre-Tfh sample.

**Supplementary table 2: *Dilution factors for flow cytometry antibodies***

| **Antibody** | **Manufacturer** | **Volume per test (μl)** | **Dilution factor** |
| --- | --- | --- | --- |
| CD45RA AF700 | BD Pharmingen | 5 | 1:50 |
| CD20 APC-Cy7 | BD Biosciences | 20 | 1:10 |
| CD3 PERCP-Cy5.5 | BD Biosciences | 10 | 1:25 |
| PD-1 BV421 | BD Biosciences | 5 | 1:50 |
| CD4 BV605 | BD Biosciences | 5 | 1:50 |
| CXCR5 PE/Dazzle594 | BioLegend | 5 | 1:50 |

**Supplementary table 3: *Numbers of cells per sample and per cluster with gene expression and TCR data***

**Supplementary table 4: *Cluster defining differentially expressed genes***

| **Gene** | **Function** | **Cluster** | **Reference** |
| --- | --- | --- | --- |
| *GZMK* | Encodes Granzyme K protein. | Cytotoxic | [^113^](#_ENREF_113)^,^ [^114^](#_ENREF_114) |
| *CCL5* | Encodes chemokine ligand 5 (CCL5), contained in cytoplasmic storage vesicles of cytotoxic cells and reported to enhance CTL-mediated cytolysis and triggers CXCR4 expression, promoting CTL migration along CXCL12 gradient. | Cytotoxic | [^115-117^](#_ENREF_115) |
| *CCL4* | Encodes chemokine ligand 4 (CCL4), reported to be significantly correlated with expression of CTL markers and promoter of CTL migration in conjunction with CCL5. Increased expression of *CCL4* was also observed in cytolytic CD4^+^ T cells. | Cytotoxic | [^115^](#_ENREF_115)^,^ [^118^](#_ENREF_118)^,^ [^119^](#_ENREF_119) |
| *SLAMF7* | Involved in cellular activation and differentiation and agonistic engagement of the SLAMF7 receptor has been shown to enhance cytotoxicity of tumour-specific CD4^+^ T cells. | Cytotoxic | [^118^](#_ENREF_118) |
| *GZMA* | Encodes Granzyme A protein. | Cytotoxic | [^113^](#_ENREF_113)^,^ [^114^](#_ENREF_114) |
| *EOMES* | Transcription factor Eomesodermin, shown to drive IFN-γ secretion and marks a cytotoxic signature. | Cytotoxic | [^120^](#_ENREF_120)^,^ [^121^](#_ENREF_121) |
| *MALAT1* | A long non-coding RNA that plays a role in transcriptional regulation of genes. | Resting | [^122^](#_ENREF_122)^,^ [^123^](#_ENREF_123) |
| *MT-ND2* | Essential for catalytic activity and assembly of complex 1. | Resting | [^124^](#_ENREF_124) |
| *MT-ATP6* | Involved in mitochondrial ATP synthesis coupled proton transport. | Resting | [^125^](#_ENREF_125) |
| *SELL* | Encodes CD62L and mediates adherence of lymphocytes to endothelial cells of high endothelial venules, thereby mediating cellular migration. | Activated migrating; B cell interacting Tfh | [^47^](#_ENREF_47)^,^ [^126^](#_ENREF_126) |
| *CCR7* | Promotes memory T cell homing to the LN paracortex and regulates T cell homeostasis within the LN. | Activated migrating | [^127^](#_ENREF_127)^,^ [^128^](#_ENREF_128) |
| *GPR183* | Involved in positioning of activated T cells at the B cell follicle border, promoting Tfh cell differentiation and cellular interactions. | Activated migrating; B cell interacting Tfh | [^22^](#_ENREF_22)^,^ [^129^](#_ENREF_129) |
| *LAT* | Required for TCR mediated signalling and cell activation. | Activated migrating | [^130^](#_ENREF_130)^,^ [^131^](#_ENREF_131) |
| *ANXA1* | Regulated activated T cell differentiation and proliferation by enhancing signalling cascades triggered by activation. | Activated migrating | [^132^](#_ENREF_132)^,^ [^133^](#_ENREF_133) |
| *CD69* | Marker of early T cell activation. | Activated migrating | [^134^](#_ENREF_134)^,^ [^135^](#_ENREF_135) |
| *TXNIP* | Inhibits mTORC1 via inhibition of proteasomal degradation of DDIT4. | Activated migrating | [^136^](#_ENREF_136) |
| *DUSP1* | The encoded protein dephosphorylates MAP kinase MAPK1/ERK2 and plays a role in negative regulation of cell proliferation. | Activated migrating | [^137^](#_ENREF_137)^,^ [^138^](#_ENREF_138) |
| *OTUB1* | Regulator of T cell anergy via RNF128/GRAIL interaction. | B cell interacting Tfh | [^139^](#_ENREF_139) |
| *PDCD1* | Encodes the canonical Tfh marker, PD-1. An inhibitory marker expressed on antigen-activated T cells that is involved in induction and maintenance of immune tolerance to self. | B cell interacting Tfh | [^140^](#_ENREF_140) |
| *MKI67* | Encodes Ki67 protein which is necessary for cellular proliferation. Prevents chromosome collapse and enables chromosome motility and is required for maintenance of individual mitotic chromosome within the cytoplasm following nuclear envelope disassembly. | Proliferating | [^141^](#_ENREF_141) |
| *UBE2C* | Controls progression through mitosis in its role as an essential factor of the anaphase promoting complex/cyclosome (APC/C). | Proliferating | [^142^](#_ENREF_142)^,^ [^143^](#_ENREF_143) |
| *CENPA* | Required for recruitment and assembly of kinetochore proteins, subsequently playing a critical role in chromosome segregation, cytokinesis, and mitosis progression. | Proliferating | [^144-147^](#_ENREF_144) |
| *CDCA8* | Essential in chromosome alignment and segregation, and microtubule stabilisation and spindle assembly. | Proliferating | [^148^](#_ENREF_148) |
| *CCNB2* | Encodes B-type cyclins which are essential components of the cell cycle regulatory machinery. Essential for control at the G2/M phase transition. | Proliferating | [^149^](#_ENREF_149)^,^ [^150^](#_ENREF_150) |
| *CDK1* | Essential for G1/S and G2/M phase transitions. | Proliferating | [^151^](#_ENREF_151)^,^ [^152^](#_ENREF_152) |
| *CCNA2* | Essential for G1/S and G2/M phase transitions. | Proliferating | [^153^](#_ENREF_153) |
| *CCNB1* | Essential for control of the G2/M phase transition. | Proliferating | [^149^](#_ENREF_149) |
| *PRC1* | Essential for the control of the spatiotemporal formation of the midzone during cytokinesis. Present in high levels during the S and G2/M phases of mitosis. | Proliferating | [^154^](#_ENREF_154)^,^ [^155^](#_ENREF_155) |
| *RPL21P16* | Ribosomal protein L21 pseudogene 16 | All clusters |  |
| *TSC22D3* | Protects T cells from IL2 deprivation-induced apoptosis and plays an important role in the anti-inflammatory and immunosuppressive effects of glucocorticoids. | All clusters | [^156^](#_ENREF_156) |
| *JUNB* | Transcription factor involved in positive regulation of transcription by RNA polymerase II. | Resting and Activated migrating | [^157^](#_ENREF_157) |
| *IL7R* | Plays a critical role in VDJ recombination during lymphocyte development. | All clusters | [^158^](#_ENREF_158)^,^ [^159^](#_ENREF_159) |
| *RPLP2* | Encodes a ribosomal phosphoprotein that plays an important role in the elongation step of protein synthesis. | Activated migrating | [^160^](#_ENREF_160) |
| *NDUFA13* | Required for mitochondrial membrane respiratory chain complex 1 assembly and electron transfer activity. | B cell interacting Tfh and Proliferating | [^161^](#_ENREF_161) |
| *CIAO2B* | Part of the mitotic spindle-associated MMXD complex and plays a role in chromosome segregation and progression of mitosis. | B cell interacting Tfh | [^162^](#_ENREF_162)^,^ [^163^](#_ENREF_163) |
| *ILF2* | Protein encoded by this gene is required for T cell expression of IL2 gene. Forms a stable dimer with ILF3 and plays key role in several biological processes e.g. cell growth. | B cell interacting Tfh | [^164^](#_ENREF_164) |
| *AURKB* | Forms part of the chromosomal passenger complex that acts as a key regulator of mitosis. | Proliferating | [^165-167^](#_ENREF_165) |
| *STMN1* | Involved in regulation of microtubule filament systems by promoting disassembly. | Proliferating | [^168^](#_ENREF_168) |

**Supplementary table 5: *TCR αβ clones across transcriptionally distinct clusters***

**Supplementary table 6: *Curated T cell specific gene set***

SupportingInformation_TableS6.xlsx
